# Measuring Adult Mortality Using Sibling Survival: A New Analytical Method and New Results for 44 Countries, 1974–2006

**DOI:** 10.1371/journal.pmed.1000260

**Published:** 2010-04-13

**Authors:** Ziad Obermeyer, Julie Knoll Rajaratnam, Chang H. Park, Emmanuela Gakidou, Margaret C. Hogan, Alan D. Lopez, Christopher J. L. Murray

**Affiliations:** 1 Institute for Health Metrics and Evaluation, University of Washington, Seattle, Washington, United States of America; 2 Department of Emergency Medicine, Brigham and Women's Hospital, Boston, Massachusetts, United States of America; 3 Mount Sinai School of Medicine, New York, New York, United States of America; 4 School of Population Health, The University of Queensland, Brisbane, Queensland, Australia; London School of Hygiene and Tropical Medicine, United Kingdom

## Abstract

**Background:**

For several decades, global public health efforts have focused on the development and application of disease control programs to improve child survival in developing populations. The need to reliably monitor the impact of such intervention programs in countries has led to significant advances in demographic methods and data sources, particularly with large-scale, cross-national survey programs such as the Demographic and Health Surveys (DHS). Although no comparable effort has been undertaken for adult mortality, the availability of large datasets with information on adult survival from censuses and household surveys offers an important opportunity to dramatically improve our knowledge about levels and trends in adult mortality in countries without good vital registration. To date, attempts to measure adult mortality from questions in censuses and surveys have generally led to implausibly low levels of adult mortality owing to biases inherent in survey data such as survival and recall bias. Recent methodological developments and the increasing availability of large surveys with information on sibling survival suggest that it may well be timely to reassess the pessimism that has prevailed around the use of sibling histories to measure adult mortality.

**Methods and Findings:**

We present the Corrected Sibling Survival (CSS) method, which addresses both the survival and recall biases that have plagued the use of survey data to estimate adult mortality. Using logistic regression, our method directly estimates the probability of dying in a given country, by age, sex, and time period from sibling history data. The logistic regression framework borrows strength across surveys and time periods for the estimation of the age patterns of mortality, and facilitates the implementation of solutions for the underrepresentation of high-mortality families and recall bias. We apply the method to generate estimates of and trends in adult mortality, using the summary measure _45_ *q*_15_—the probability of a 15-y old dying before his or her 60th birthday—for 44 countries with DHS sibling survival data. Our findings suggest that levels of adult mortality prevailing in many developing countries are substantially higher than previously suggested by other analyses of sibling history data. Generally, our estimates show the risk of adult death between ages 15 and 60 y to be about 20%–35% for females and 25%–45% for males in sub-Saharan African populations largely unaffected by HIV. In countries of Southern Africa, where the HIV epidemic has been most pronounced, as many as eight out of ten men alive at age 15 y will be dead by age 60, as will six out of ten women. Adult mortality levels in populations of Asia and Latin America are generally lower than in Africa, particularly for women. The exceptions are Haiti and Cambodia, where mortality risks are comparable to many countries in Africa. In all other countries with data, the probability of dying between ages 15 and 60 y was typically around 10% for women and 20% for men, not much higher than the levels prevailing in several more developed countries.

**Conclusions:**

Our results represent an expansion of direct knowledge of levels and trends in adult mortality in the developing world. The CSS method provides grounds for renewed optimism in collecting sibling survival data. We suggest that all nationally representative survey programs with adequate sample size ought to implement this critical module for tracking adult mortality in order to more reliably understand the levels and patterns of adult mortality, and how they are changing.

*Please see later in the article for the Editors' Summary*

## Abstract 2

Julie Rajaratnam and colleagues describe a novel method, called the Corrected Sibling Survival method, to measure adult mortality in countries without good vital registration by use of histories taken from surviving siblings.

## Editors' Summary

**Background:**

Governments and international health agencies need accurate information on births and deaths in populations to help them plan health care policies and monitor the effectiveness of public-health programs designed, for example, to prevent premature deaths from preventable causes such as tobacco smoking. In developed countries, full information on births and deaths is recorded in “vital registration systems.” Unfortunately, very few developing countries have complete vital registration systems. In most African countries, for example, less than one-quarter of deaths are counted through vital registration systems. To fill this information gap, scientists have developed several methods to estimate mortality levels (the proportion of deaths in populations) and trends in mortality (how the proportion of deaths in populations changes over time) from data collected in household surveys and censuses. A household survey collects data about family members (for example, number, age, and sex) for a national sample of households randomly selected from a list of households collected in a census (a periodic count of a population).

**Why Was This Study Done?:**

To date, global public-health efforts have concentrated on improving child survival. Consequently, methods for calculating child mortality levels and trends from surveys are well-developed and generally yield accurate estimates. By contrast, although attempts have been made to measure adult mortality using sibling survival histories (records of the sex, age if alive, or age at death, if dead, of all the children born to survey respondents' mothers that are collected in many household surveys), these attempts have often produced implausibly low estimates of adult mortality. These low estimates arise because people do not always recall deaths accurately when questioned (recall bias) and because families that have fallen apart, possibly because of family deaths, are underrepresented in household surveys (selection bias). In this study, the researchers develop a corrected sibling survival (CSS) method that addresses the problems of selection and recall bias and use their method to estimate mortality levels and trends in 44 developing countries between 1974 and 2006.

**What Did the Researchers Do and Find?:**

The researchers used a statistical approach called logistic regression to develop the CSS method. They then used the method to estimate the probability of a 15-year-old dying before his or her 60th birthday from sibling survival data collected by the Demographic and Health Surveys program (DHS, a project started in 1984 to help developing countries collect data on population and health trends). Levels of adult mortality estimated in this way were considerably higher than those suggested by previous analyses of sibling history data. For example, the risk of adult death between the ages of 15 and 60 years was 20%–35% for women and 25%–45% for men living in sub-Saharan African countries largely unaffected by HIV and 60% for women and 80% for men living in countries in Southern Africa where the HIV epidemic is worst. Importantly, the researchers show that their mortality level estimates compare well to those obtained from vital registration data and other data sources where available. So, for example, in the Philippines, adult mortality levels estimated using the CSS method were similar to those obtained from vital registration data. Finally, the researchers used the CSS method to estimate mortality trends. These calculations reveal, for example, that there has been a 3–4-fold increase in adult mortality since the late 1980s in Zimbabwe, a country badly affected by the HIV epidemic.

**What Do These Findings Mean?:**

These findings suggest that the CSS method, which applies a correction for both selection and recall bias, yields more accurate estimates of adult mortality in developing countries from sibling survival data than previous methods. Given their findings, the researchers suggest that sibling survival histories should be routinely collected in all future household survey programs and, if possible, these surveys should be expanded so that all respondents are asked about sibling histories—currently the DHS only collects sibling histories from women aged 15–49 years. Widespread collection of such data and their analysis using the CSS method, the researchers conclude, would help governments and international agencies track trends in adult mortality and progress toward major health and development targets.

**Additional Information:**

Please access these Web sites via the online version of this summary at http://dx.doi.org/10.1371/journal.pmed.1000260.

- This study and two related *PLoS Medicine* Research Articles by Rajaratnam et al. and by Murray et al. are further discussed in a *PLoS Medicine* Perspective by Mathers and Boerma
- Information is available about the Demographic and Health Surveys
- The Institute for Health Metrics and Evaluation makes available high-quality information on population health, its determinants, and the performance of health systems
- Grand Challenges in Global Health provides information on research into better ways for developing countries to measure their health status
- The World Health Organization Statistical Information System (WHOSIS) is an interactive database that brings together core health statistics for WHO member states, including information on vital registration of deaths; the WHO Health Metrics Network is a global collaboration focused on improving sources of vital statistics

## Introduction

For several decades, global public health efforts have focused on the development and application of disease control programs to improve child survival in developing countries. Technologies for preventing and successfully treating the leading causes of death in children are available, and their increasing effective use is leading to continuing declines in child mortality. The need to reliably monitor the impact of such intervention programs in countries has led to significant advances in demographic methods and data sources, particularly from large-scale. cross-national survey programs such as the Demographic and Health Surveys (DHS) [1]–[3]. As a consequence, levels and trends of child mortality are reasonably well understood in most countries [4],[5].

Despite the impetus from the global HIV epidemic and increasing global concern with avoiding premature adult death from tobacco and other established and modifiable causes of disease, we remain remarkably ignorant about current levels of adult mortality in the majority of developing countries and how they are changing. Vital registration systems are generally incomplete in most developing countries. Compilations of adult mortality estimates are routinely made by international organizations such as the United Nations [6], the World Health Organization [7], and the World Bank [8], but these are largely model-based, and there is considerable uncertainty about whether their demographic assumptions are accurate in contemporary developing populations. This situation has led to the development of alternative measurement strategies for adult mortality [1]–[3].

The availability of large datasets with information on adult survival from censuses and household surveys offers an important opportunity to improve our knowledge about levels and trends in adult mortality in countries without good vital registration. Methods to use such data can be broadly classified as either “indirect”—relying on demographic models and simple summary statistics from respondents—or “direct,” which entail fewer analytical assumptions but are more onerous as detailed time-location information must be collected. Hill and Trussell discussed spousal survival and parental survival as indirect estimators of adult mortality in 1977; in the same paper, they also introduced and argued for the wider use of sibling survival data to estimate adult mortality [9]. More than 10 y following, the need to reliably estimate maternal mortality led to the development of the indirect sisterhood method [10] and the eventual inclusion of a full sibling history in many household surveys [11], allowing for the direct estimation of adult mortality.

Sibling survival is especially appealing for the measurement of adult mortality because a single respondent can provide information on a potentially large number of siblings, providing estimates of mortality levels by age and sex at different periods of time prior to the survey [1]. However, there are numerous potential biases that have limited the use of these data. Families that have disintegrated because of discord or death will be underrepresented in population surveys, implying that mortality measures calculated from surveys will be biased downward. It is also likely that respondents will fail to recall some deaths of siblings, especially those that occurred several years prior to the survey, or in cases where the respondent has not had recent contact with his or her siblings [11],[12].

Despite these concerns, since the incorporation of the maternal mortality module into the DHS program [13], several authors have used these complete sibling histories to directly estimate adult mortality. Bicego [11] examined adult mortality in the context of the HIV/AIDS epidemic in six of the early sibling history modules from sub-Saharan Africa. In another study motivated by the HIV/AIDS epidemic, Timaeus [14] showed that sibling histories can capture plausible trends and produce estimates similar in level to indirect estimates via parental survival.

Other studies have been less optimistic about the quality of mortality data produced from sibling histories. Stanton et al. [15], in examining the data quality of the DHS maternal mortality module, conclude that sibling histories seem to produce an underestimation of adult mortality, an effect more pronounced among women. Gakidou et al. [1] reach a similar conclusion, noting that while analogous birth history data produce child mortality estimates consistent with modeled UN estimates, adult mortality numbers from sibling histories are substantially lower.

The most comprehensive analysis of sibling history data to date was carried out by Timaeus and Jasseh [12]. This regression-based analysis quantified trends in adult mortality in 23 sub-Saharan countries using 26 surveys. The evidence from sibling history data is consistent with parental survival estimates, but there is strong evidence of substantial recall bias leading to an exaggerated rate of mortality rise.

Recent methodological work has addressed the issue of selection bias in sibling survival data [16], a persistent concern expressed in the literature. Recall bias is another limitation in the use of sibling history data, but the use of consecutive surveys to evaluate the magnitude of recall bias has been explored in the estimation of both child mortality [17] and fertility [18],[19].

Building on this literature, in this article we develop new methods to adjust for the underestimation of mortality arising from the recall of deaths, adapt the Gakidou-King (GK) approach to correct for selection bias, and propose a new correction for the downward bias resulting from zero-survivor families. We collectively refer to this new set of methods as the Corrected Sibling Survival (CSS) method. We have applied the CSS method to sibling survival data from 83 surveys in 44 countries, primarily in Africa, where uncertainty about true levels and trends in adult mortality has been greatest.

## Methods

### Data

We used data from the DHS program. The standard DHS instrument, with nationally representative sample sizes ranging from 3,000 to 90,000 women of reproductive age (typically 15–49 y), is widely used for demographic estimation [20],[21]. As of June 25, 2009, 86 surveys in 46 countries had incorporated a sibling history (also known as the maternal mortality) module. This module collects information from the respondent on each sibling born to the same mother, including sex, age, whether alive, and if dead, the date of the death. Table S1 summarizes the characteristics of the sibling history modules from these surveys with various measures of survey quality.

In addition to the DHS, we used supplementary sources of data to categorize country-periods into groups of similar age patterns (explained in detail below). For this purpose, we used HIV seroprevalence estimates from UNAIDS historical time series [22]. These seroprevalence numbers are based on data from antenatal clinics, population-based surveys, and models that synthesize the available data. They are the most comprehensive estimates of historical HIV seroprevalence available. We identified country-periods with a substantial history of war using combined data from the PRIO [23] war databases for battle deaths, one-sided war deaths, and nonstate war deaths. Finally, we categorized country-periods using estimates of child mortality from Murray and colleagues' analysis of multiple data sources [4].

We have excluded three DHS from our analysis, and these are not included in Table S1. On the basis of published reports of poor data quality in the sibling history module, we excluded the 1999 Nigeria survey [24]. We excluded the 1997 Jordan survey because HIV seroprevalence time series data were not available. We also excluded the 1996 Nepal survey because ages and dates are not coded consistently.

### Data Structure

The sibling history data are structured such that there is one observation per respondent, with the entire collection of siblings born to the same mother (“sibship”) recorded within that observation. The application of the CSS method requires substantial restructuring of the data. We first reshaped each dataset so that there is one observation per sibling, including one observation for the respondent. We then reshaped the data again, so that one observation refers to one person-year of one sibling. In this data structure, every member of a sibship received an observation for every year that they were alive and an observation for the year in which they died, if they died. Only years where observation is complete for all siblings were used; in other words, we truncated the data to the last complete calendar year before the survey. Each observation also includes information on the sex of the sibling, the calendar year of the observed person-year, the age of the sibling in that calendar year, and the survival status of the sibling within that year. Since the DHS only interviews women up to 49 y of age, the data become increasingly sparse for the older age groups as we trace the sibling records back in time. To avoid small number problems, we only used sibling history data up to 15 y prior to the survey year.

### Dependent Variable

The dependent variable is a dummy variable indicating the sibling's survival status during the particular person-year of observation. A value of 1 indicates that the individual died during the year of observation, while a value of 0 indicates that she or he was alive at the end of the year of observation.

### Independent Variables

There are three groups of independent variables included in our model: (1) country-period effects, (2) age patterns, and (3) recall bias.

First, we included a dummy variable for each country and 5-y calendar period. This step allows the level of mortality to vary nonparametrically by country and time-period. We experimented with both 2-y and 5-y time periods and opted for 5-y time periods because it was evident that the 2-y periods did not have enough observations to generate stable rates over time. The one exception to the 5-y periods is the Rwanda genocide. Because mortality rates were quite different in the surrounding years and because so many deaths were reported in 1993 and 1994, to generate a stable 2-y estimate, we kept 1993–1994 as a 2-y period in Rwanda and created 5-y periods for the rest of the data.

Second, we modeled stable age patterns across sets of countries because each survey has insufficient person-years of observation to generate a stable pattern of mortality by age. Given the demographic and epidemiology literature, we expect that in the absence of major shocks such as war or HIV, death rates between the ages of 15 and 60 y will change in a consistent way with respect to age [25]. The slope of this relationship also tends to get steeper as general mortality levels decline (this can be seen by plotting the age patterns of mortality for sequential time periods for a country with good historical vital registration data like Sweden's [26]). Further, we know that major war events tend to lead to disproportionate increases in mortality at younger adult ages. Finally, the HIV epidemic characteristically increases mortality at younger adult ages and has a smaller effect at older ages [14].

With these expectations of differences in age patterns and also to test the sensitivity of the final estimates to different ways of modeling age patterns, we have implemented four different approaches to modeling age patterns across the country-periods in the sibling history data. Model 1 uses a constant age-pattern of mortality across all country-periods. The other three models group country-periods into four different classes for modeling age patterns. Model 2 divides country-periods into four groups on the basis of HIV prevalence (0%–1.9%, 2%–6.9%, 7%–11.9%, and ≥12%). Model 3 divides country-periods into one group with a history of substantial war, and the remainder into three groups on the basis of general levels of mortality as captured by levels of child mortality. The three groups of child mortality were determined by tertiles of the child mortality distribution for the country-periods in the sibling history dataset. Model 4 divides country-periods into four groups: those with a history of substantial war, those with high HIV (seroprevalence greater than 7%), and for the remainder, low (bottom 2 tertiles) and high (top tertile) levels of child mortality. For models 2 and 4, we use 2008 UNAIDS historical estimates of HIV seroprevalence but classify on the basis of the 5-y lag of HIV seroprevalence, which accounts for the lag between HIV incidence and mortality [27]. For models 3 and 4, country-periods with greater than 0.75 recorded war deaths per 1,000 population are included in the war grouping, as are the following country-periods, since we observe in the raw data a continuation of war-like age patterns for some years post-conflict.

For models 2 through 4, the implementation of the models involved constructing four dummy variables (denoted *H*_0_, *H*_1_, *H*_2_, and *H*_3_) to indicate to which age-pattern group within that particular model an observation belonged. We then interacted these four dummy variables with a set of dummy variables for each 5-y age group (15–19, 20–24, …, 55–59). By including a set of dummy variables representing each age group in the model, we borrowed strength across all surveys to inform the age pattern of mortality without imposing a model-based age structure a priori. By interacting the age dummies with the pattern-group dummies, we allowed the age pattern of mortality to vary according to the above-mentioned criteria used to establish each set of age-pattern groups.

Third, we included a continuous variable—time prior to the survey (*TiPS*)—which measures how many years prior to the survey each person-year of observation occurred. For example, if a respondent to a survey carried out in the year 2000 reported on the death of a sibling in 1990, the value of *TiPS* would be 10 for that observation. Several previous studies have raised the possibility that respondents may omit reporting some sibling deaths [12],[15],[16],[28]. The *TiPS* variable empirically measures recall bias and can be used to correct for it. Intuitively, *TiPS* captures the difference between deaths reported in the more recent periods of older surveys and deaths reported for more distant periods in more recent surveys. It can only be estimated if there is sufficient overlap of observations from different surveys for the same country-year. When modeled as dummy variables, we found the relationship of the *TiPS* values to be essentially linear with the log odds of death, so we included *TiPS* as a continuous variable in our final model. In the regression models presented below, we assumed that recall bias is the same across countries. To test this, we performed a sensitivity analysis and compared country-specific estimates of the *TiPS* coefficient with the all-country coefficient estimated in our regression model.

### Selection Bias

Sibling-year observations are weighted to address the underrepresentation of high-mortality families in population-based surveys following the general methods proposed by Gakidou and King [16]. This method (which we abbreviate GK) incorporates a family-level weight, 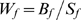, where *B_f_* is the original sibship size and *S_f_* is the number of siblings in family *f* surviving to the time of the survey. This weight algebraically corrects for the underrepresentation of high-mortality families in the survey sample. To generate the final weight for each observation, we multiplied *W_f_* by the DHS sampling weight.

The GK weights alone do not fully address the issue of selection bias. The respondents to the sibling history survey are exclusively women, so by definition the sample includes no sibships where (1) the siblings are all men, or (2) all the women have died. In the first case, we assumed that the mortality rates for men in all-male sibships are the same as mortality rates for males in sibships with one or more females. In this case, mortality rates for men will not be biased. In the second case, where all the women in some sibships have died, reweighted mortality rates that ignore this will be biased downward. Therefore, we needed to further adjust the female rates to account for this bias, which we refer to as zero-female-survivor bias (the reverse would be true if all respondents were men). Figure S1 and Text S1 show our proposed correction for the fact that the surveys do not capture sibships with zero female survivors.

### Regression Model

There is a long history of using logistic regression to model mortality/survival in epidemiology and demography [29]–[32]. We employed a logistic regression framework to directly estimate the probability of death by age, sex, time period, and country. We pooled data from all surveys and applied the following logistic regression models estimated separately for males and females:
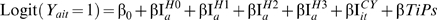
Where *Y*_ait_ indicates survival or death of an individual in age group *a*, in country *i*, at time *t*, 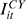 is the set of dummy variables denoting country *i* in the 5-y period designated by *t*. 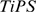 is the continuous variable representing time prior to the survey. 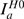 through 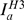 are the four sets of dummy variables indicating the 5-y age groups from 15 to 60 (*a* = 20–24, 25–29, …, 55–59, with 15–19 as the reference category) and dependent on *H*_0_, *H*_1_, *H*_2_, and *H*_3_, the criteria used to define the age patterns, summarized in Table 1.

**Table 1:** Criteria for classifying country-periods into different age-pattern groups.

Variable Notation	Model I	Model II	Model III	Model IV
***H*** **_0_**	Single pattern	0%–1.9% HIV	War	War
***H*** **_1_**	N/A	2%–6.9% HIV	Low tertile _5_ *q*_0_	≥7% HIV
***H*** **_2_**	N/A	7%–11.9% HIV	Mid tertile _5_ *q*_0_	Low/mid tertiles _5_ *q*_0_
***H*** **_3_**	N/A	≥12% HIV	High tertile _5_ *q*_0_	High tertile _5_ *q*_0_

N/A, not applicable.

We computed standard errors to allow for clustering at the primary sampling unit (PSU) level. The correlation of the probability of death at the individual level (i.e., the fact that a sibling's probability of death at time *t* is likely to be correlated with his probability of death at time 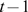) will lead to artificially low standard errors. This problem would typically be addressed by clustering standard errors at the individual level (equivalent to the concept of shared frailty in survival analysis); however, our approach of clustering errors at the higher PSU level produces even larger standard errors than clustering at the individual level, and we thus view it as a more conservative approach.

### Estimating _45_ *q*_15_

Our ultimate quantity of interest is the probability of dying between ages 15 and 60, a commonly used indicator of adult mortality that can be compared across populations. In standard demographic notation, this is referred to as _45_ *q*_15_.

We used the coefficients from the logistic regression model to predict all linear combinations of age group, time period, country, and sex. To estimate what the level of adult mortality would be in the absence of recall bias, we set the coefficient on the *TiPS* variable to zero. The inverse logit transformation of the predicted values represents the 1-y probabilities of death for each particular age group, time period, country, and sex. From these single year age-specific probabilities, we computed age-specific probabilities of survival and then 5-y estimates of _45_ *q*_15_.

### Comparing Model Fits

We compared the fits of models 1 through 4 using three metrics: (1) the root mean squared error (RMSE) comparing differences in predicted age-specific probabilities of death (_n_ *q*_x_) to observed; (2) RMSE of differences between predicted and observed _n_ *q*_x_ in log space (which weights differences across the age groups more equally); and (3) RMSE of differences between predicted and observed _45_ *q*_15_, our summary measure of adult mortality. For each metric, we ranked the performance of the model by sex and then created a summary rank score by adding the ranks for each metric across both sexes. We considered the model with the lowest summary rank score (the one that overall minimizes the differences between predicted and observed values across sexes) to have the best fit.

### Uncertainty

Following the methods outlined in King, Tomz, and Wittenberg [33], uncertainty in the model parameters used to generate estimates of _45_ *q*_15_ is captured by taking 1,000 simultaneous draws from the variance-covariance matrix of the logistic regression model and then producing 1,000 estimates of _5_ *q*_x_ for every age group and 1,000 corresponding estimates of _45_ *q*_15_ as outlined above. The 95% uncertainty interval is defined by the 25th and 976th ranked estimates of the 1,000 simulated values.

All analyses were carried out in Stata 10.1/MP [34]. Data files of final estimates and Stata code used to produce them are available from the authors upon request.

## Results

### CSS Model Results

**Figure 1:**
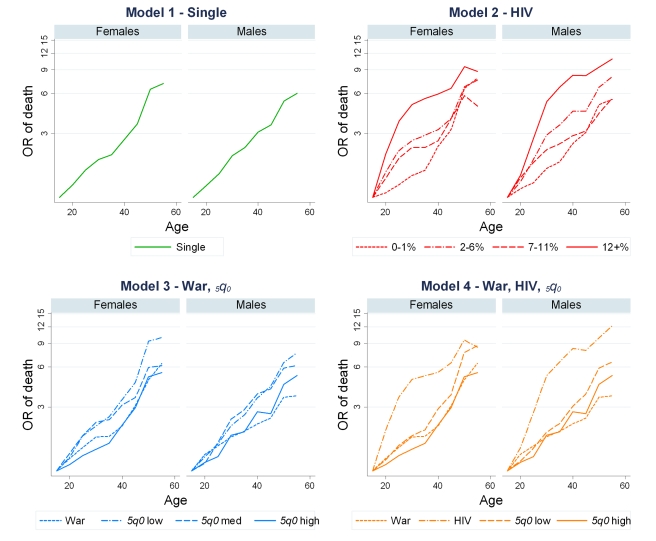
Age patterns from all four models. Model 1 groups country-periods into a single age pattern. Model 2 groups country-periods according to level of HIV seroprevalence. Model 3 groups country-periods by history of war and levels of _5_ *q*_0_, and model 4 is a hybrid model containing one group of country-periods with history of war, one group with high levels (≥7%) of HIV seroprevalence, and two groups based on _5_ *q*_0_ levels, low and high.

The RMSEs between observed and predicted values for each of the four models are shown in Table 2, along with the summary rank score for each model. The full set of regression results (including age pattern, *TiPS*, and all 175 country-period coefficients) can be found in Table S2. Figure 1 graphs each set of age patterns relative to 15–19 y olds in a given country-period. The age patterns for populations with high prevalence of HIV are similar to what is seen in South African vital registration data in recent years (comparison not shown). In models 3 and 4, the slope of the war age pattern is less steep across the age range compared to the lower _5_ *q*_0_ age patterns, and more so for males than for females, which is expected given young males tend to be the age group most susceptible to battle death in times of war. Similarly, across levels of _5_ *q*_0_, the slope of mortality over age decreases with higher levels of child mortality, again similar to what we observe in other data sources [26]. We chose model 4 as our optimal model because it minimized the differences between the empirically observed and model predicted age patterns as well as between observed and predicted _45_ *q*_15_, thus ensuring the closest fit to the data. However, the resultant estimates of _45_ *q*_15_ generated from each of the models are quite similar (correlation coefficients comparing any two models ranged from 0.9788 to 0.9932).

**Table 2:** Model fit results from applying different age-pattern groupings to sibling survival data.

Sex	Statistic	Model I	Model II	Model III	Model IV
**Females**					
	**RMSE of _n_** ***q*** **_x_**	0.02956	0.02836	0.02891	0.02902
	**Rank**	4	1	2	3
	**RMSE of ln(_n_** ***q*** **_x_)**	0.42823	0.40932	0.41908	0.41218
	**Rank**	4	1	3	2
	**RMSE of _45_** ***q*** **_15_**	0.06572	0.06591	0.06361	0.06507
	**Rank**	3	4	1	2
**Males**					
	**RMSE of _n_** ***q*** **_x_**	0.04121	0.04071	0.03948	0.03931
	**Rank**	4	3	2	1
	**RMSE of ln(_n_** ***q*** **_x_)**	0.41582	0.40005	0.40547	0.39533
	**Rank**	4	2	3	1
	**RMSE of _45_** ***q*** **_15_**	0.09139	0.09147	0.08660	0.08757
	**Rank**	3	4	1	2
**Both sexes**	**Summary rank score**	22	15	12	11

RMSE of: differences between model-predicted age-specific probabilities of death (_n_ *q*_x_) and observed _n_ *q*_x_, differences in predicted and observed _n_ *q*_x_ in log space, and differences in predicted and observed _45_ *q*_15_ are shown. Models are ranked for each metric of fit within each sex, with 1 being best. The summary rank score is the sum of the ranks for each metric, across both sexes.

**Figure 2:**
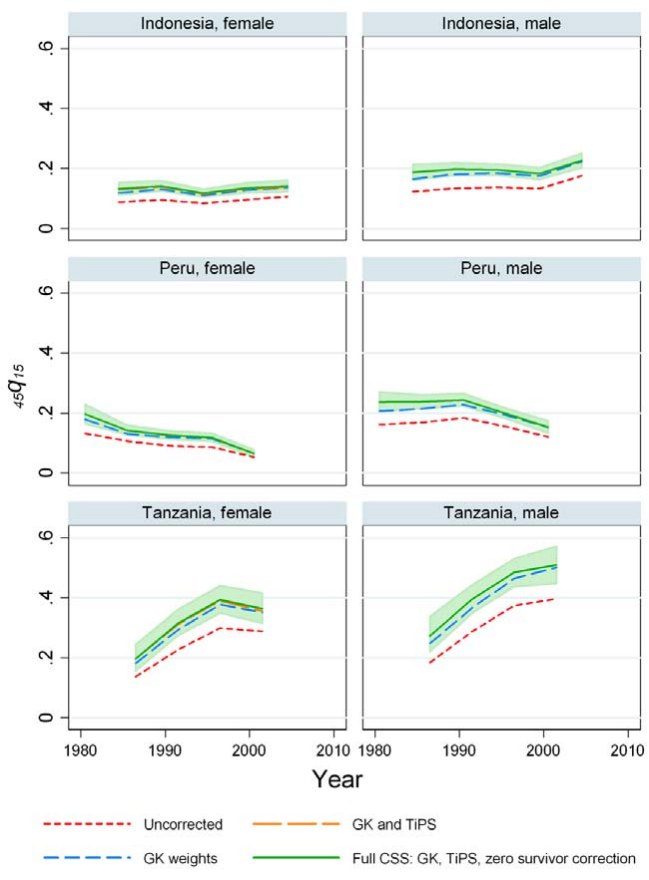
A step-by-step look at each of the adjustments in the CSS method: Indonesia, Peru, and Tanzania. The effects on mortality estimates of the GK survival bias adjustment, adjusting for recall, and the zero-female-survivor correction are shown.

**Figure 3:**
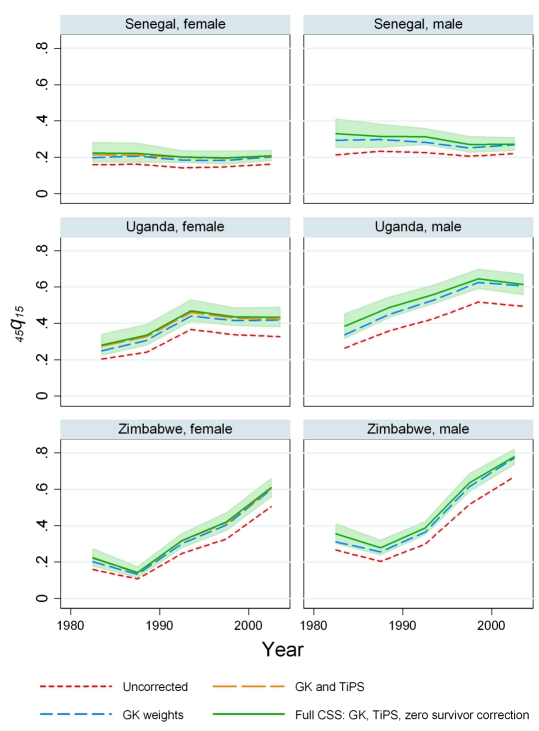
A step-by-step look at each of the adjustments in the CSS Method: Senegal, Uganda, and Zimbabwe. The effects on mortality estimates of the GK survival bias adjustment, adjusting for recall, and the zero-female-survivor correction are shown.

The impact of the three components of the CSS model on the estimates of adult mortality rates is illustrated for six countries in Figures 2 and 3. Computation of _45_ *q*_15_ using the GK weights has a major effect on measured levels of adult mortality for males and females in all countries. This indicates that underrepresentation of high mortality sibships is an important consideration when analyzing sibling survival data. On average, the GK weights raise the estimated _45_ *q*_15_ by 27%, ranging from 0% to 66% across country-periods.

**Figure 4:**
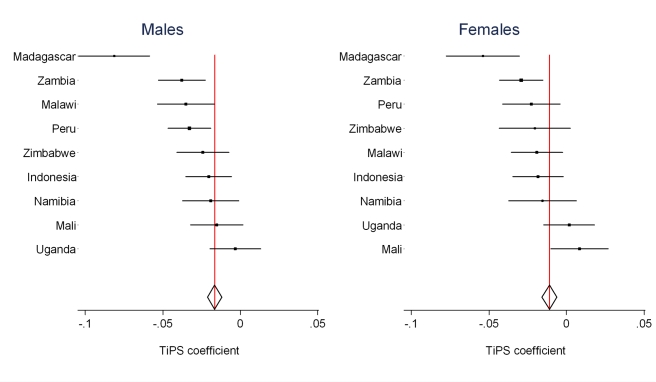
Country-specific estimates of recall bias as measured by the *TiPS* coefficient compared to the overall estimate from CSS model (red line).

Regression results for the *TiPS* variable are summarized in Table 3. For males, the all-country coefficient is −0.0165, representing a 1.64% decrease in reported deaths for each additional year prior to the survey. For females, the annual decrease is lower at 1.09%. We have also estimated the value of the *TiPS* coefficient separately for each country for the nine countries where three or more surveys with sibling history modules are available. These results are summarized in Figure 4 and show that the estimated decline in deaths reported due to recall bias varied from −0.85% (Mali females) to 7.8% (Madagascar males) across this set of countries. Of the 18 single country estimates of recall, six are statistically significantly different from the mean effect across all countries. Figures 2 and 3 illustrate how inclusion of the *TiPS* variable in the model leads to higher estimates of adult mortality, especially in time periods further removed from the survey year. Note that for some countries, the magnitude of the *TiPS* adjustment does not increase over time prior to the most recent survey. This result happens because the correction is applied to all observations from all surveys, not just the most recent. Thus, the overall correction is a function of the total number of surveys in a country and the spacing of those surveys.

**Table 3:** *TiPS* regression coefficients for countries with three or more surveys and percent decline in mortality rates per year prior to the survey attributable to recall bias.

Country	Females		Males	
	Coefficient	Percent Annual Decline	Coefficient	Percent Annual Decline
**Indonesia**	−0.0184	1.82	−0.0204	2.02
**Madagascar**	−0.0539	5.25	−0.0813	7.81
**Malawi**	−0.0191	1.89	−0.0350	3.44
**Mali**	0.0084	−0.84	−0.0152	1.51
**Namibia**	−0.0155	1.54	−0.0190	1.88
**Peru**	−0.0226	2.24	−0.0329	3.24
**Uganda**	0.0017	−0.18	−0.0032	0.32
**Zambia**	−0.0292	2.88	−0.0378	3.70
**Zimbabwe**	−0.0204	2.02	−0.0242	2.39
**All countries**	−0.0110	1.09	−0.0165	1.64

Corrections for sibships where all females have died lead to much more modest changes in the estimated rates of adult female mortality. Table 4 summarizes the magnitude of corrections to the age-specific probabilities of death for each country, ranging from 1.0% to 4.5%. The resulting changes to estimated levels of _45_ *q*_15_ range from 0.2% to 4.0%. While conceptually important, these corrections to the estimated levels of adult mortality are small. Overall, the combined effect of the GK weights, the *TiPS* recall bias correction, and the correction for missing female sibships leads to a profound increase in adult mortality rates estimated from sibling histories.

**Table 4:** Percent of deaths missing due to zero-female-survivors and the factor by which estimates of _n_ *q*_x_ are corrected upward as a result.

Country	Percent Zero-Female-Survivor Correction to _n_ *q*_x_
Benin	2.19
Bolivia	2.61
Brazil	1.27
Burkina Faso	2.86
Cambodia	3.18
Cameroon	2.75
Central African Republic	4.48
Chad	4.06
Congo (Dem. Rep.)	1.90
Congo (Rep.)	2.40
Côte d'Ivoire	2.22
Dominican Republic	1.89
Eritrea	4.29
Ethiopia	3.65
Gabon	2.14
Ghana	1.72
Guatemala	1.99
Guinea	4.11
Haiti	2.59
Indonesia	2.59
Kenya	1.23
Lesotho	2.65
Liberia	1.86
Madagascar	2.33
Malawi	2.49
Mali	3.25
Mauritania	4.08
Morocco	1.39
Mozambique	4.05
Namibia	2.08
Nepal	3.59
Niger	3.14
Peru	1.34
Philippines	1.01
Rwanda	4.06
Senegal	2.99
South Africa	2.97
Sudan	3.01
Swaziland	2.43
Togo	2.16
Uganda	3.04
United Republic of Tanzania	2.29
Zambia	2.39
Zimbabwe	1.47

### Levels of Mortality in 44 Countries

**Figure 5:**
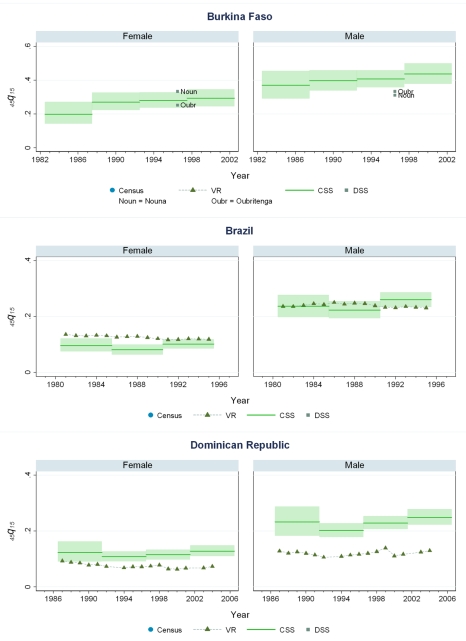
Estimates of _45_ *q*_15_ from the CSS method compared to estimates generated from vital registration, DSS, and census household death estimates: Burkina Faso, Brazil, Dominican Republic.

Figures 5–10 show CSS estimates of adult mortality for select countries using model 4; the same graphs for all countries included in our study can be found in Figure S2. Table S3 also provides estimates of *_45_q_15_* from all four models.

**Figure 6 pmed-1000260-g006:**
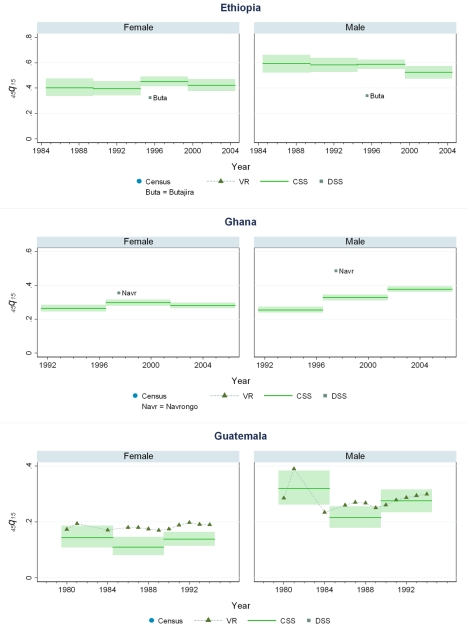
Estimates of _45_ *q*_15_ from the CSS method compared to estimates generated from vital registration, DSS, and census household death estimates: Ethiopia, Ghana, Guatemala.

**Figure 7 pmed-1000260-g007:**
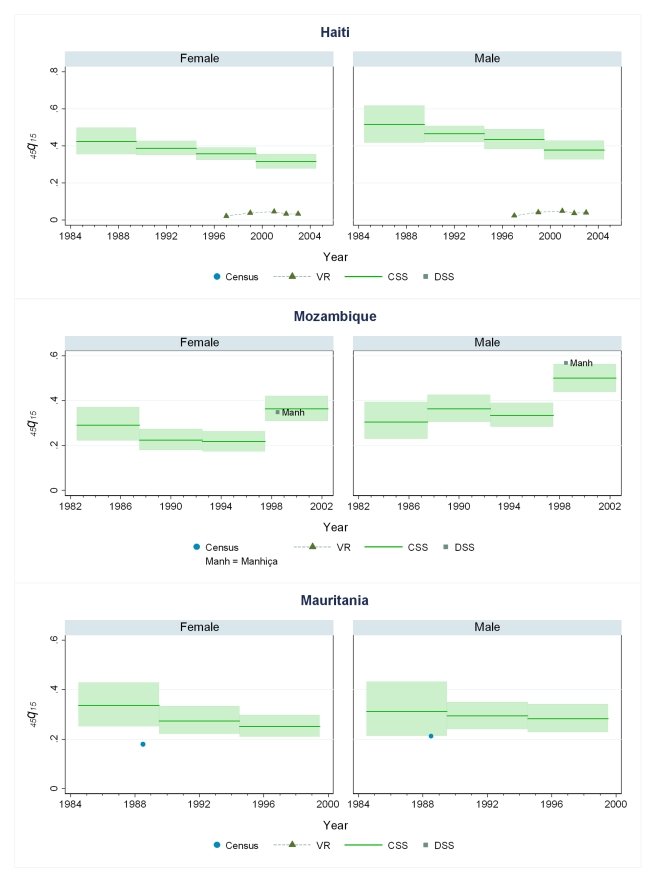
Estimates of _45_ *q*_15_ from the CSS method compared to estimates generated from vital registration, DSS, and census household death estimates: Haiti, Mozambique, Mauritania.

**Figure 8 pmed-1000260-g008:**
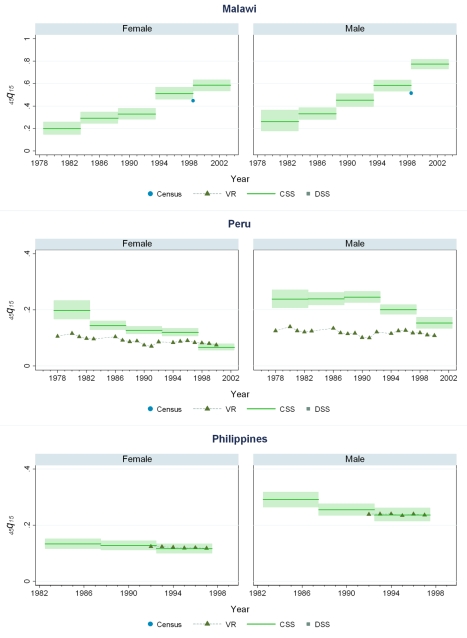
Estimates of _45_ *q*_15_ from the CSS method compared to estimates generated from vital registration, DSS, and census household death estimates: Malawi, Peru, The Philippines.

**Figure 9 pmed-1000260-g009:**
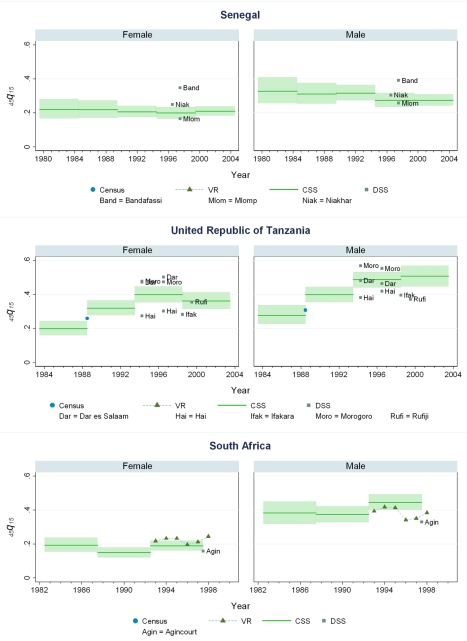
Estimates of _45_ *q*_15_ from the CSS method compared to estimates generated from vital registration, DSS, and census household death estimates: Senegal, Tanzania, South Africa.

**Figure 10:**
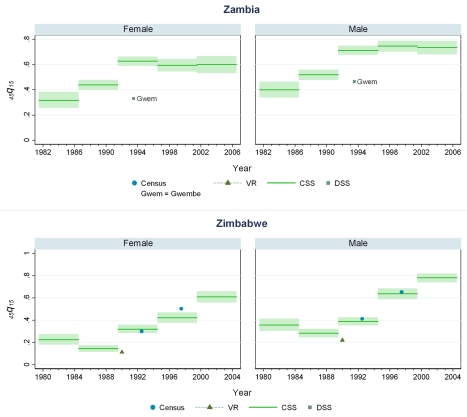
Estimates of _45_ *q*_15_ from the CSS method compared to estimates generated from vital registration, DSS, and census household death estimates: Zambia, Zimbabwe.

Our findings suggest that levels of adult mortality prevailing in many developing countries are substantially higher than previously suggested by analyses of sibling history data [1],[12]. In sub-Saharan African populations largely unaffected by HIV, we estimate the risk of death between ages 15 and 60 y to be 20%–35% for females and 25%–45% for males, though considerable heterogeneity exists among countries. In Southern African countries where the HIV epidemic has been most pronounced, rates are uniformly and strikingly high: at current rates, as many as eight out of ten men alive at age 15 y will be dead by age 60, as will as many as six out of ten women. At the height of the Rwandan genocide in 1993–1994, the probability of death between 15 and 60 y based on prevailing mortality rates was close to 100%, but has since declined to levels more typical of sub-Saharan Africa. The increase in adult mortality in countries with high HIV as mentioned above is notable. So also is the rise in mortality in some central and west African countries that have not been as affected by HIV. In Benin, mortality for both male and female adults has risen at some point in the last 15 y of available data, despite Benin having a relatively low prevalence of HIV among its population (less than 2% over the same time period). Mali, Liberia, and Guinea have also seen rises in adult mortality despite comparably low HIV seroprevalence rates (perhaps even leading one to question the validity of the seroprevalence estimates for these countries). For some West African populations, mortality rates appear to be lower, notably in Senegal and Niger, where the risk of adult death is around 20%–25%.

Adult mortality levels in populations of Asia and Latin America are generally lower than in Africa, particularly for women, though Haiti and Cambodia are notable exceptions where mortality risks are comparable to many countries in Africa. In all other developing countries with available sibling history data, the probability of dying between ages 15 and 60 y was typically around 10% for women and 20% for men; this is not much higher than 2001 estimated levels for more developed countries such as Argentina, Barbados, Mexico, Puerto Rico, and Venezuela [35].

### Comparison of CSS to Other Measurements of Mortality

Few countries with complete vital registration systems have included sibling survival data in national surveys. Validation of CSS thus depends on comparisons in those few countries that have vital registration data, demographic surveillance sites (DSS), and deaths in the last 12 mo collected in national censuses. Even though all of these are likely problematic comparators, Figures 5–10 compare our estimates to these three types of available data.

While vital registration is typically considered the gold standard for the measurement of mortality, data from Peru, Brazil, Guatemala, Dominican Republic, Philippines, and South Africa are likely to undercount national adult mortality rates. Data from the Dominican Republic and Peru are thought to be particularly incomplete, missing about 50% of adult deaths, while routine systems appear to be capturing the majority of deaths in countries such as Brazil, Guatemala, the Philippines, and South Africa [36]. Figure 6 shows that in Guatemala, CSS estimates and vital registration data are similar for most years, although the CSS captures higher levels of adult mortality in the early 1980s, coincident with the outbreak and intensification of conflict between the Guatemalan military and leftist guerilla forces. A similar level of concordance is seen for the Philippines (Figure 8). In Brazil, CSS results for females appear to be somewhat lower than for the vital registration, but comparable overall (Figure 5). In the Dominican Republic (Figure 5), the vital registration rates appear to be implausibly low, especially for males, while the CSS presents more realistic levels. Overall, the levels of mortality suggested by application of our methods are comparable to or higher than what is suggested from vital registration systems, which are known to generally undercount deaths in developing countries.

In some countries, DSS have been operating that capture vital events that occur in defined populations. While these sentinel sites are quite small (covering populations of 30,000 to 1 million), and are typically selected expediently rather than randomly, they nonetheless can be a useful source of information on mortality and fertility levels. Where these sites are operative in the countries in our dataset, Figures 5–10 also show the implied levels of adult mortality compared with CSS estimates. In most cases (e.g., South Africa, Tanzania, Senegal, Mozambique, Burkina Faso), DSS death rates for most time periods fall within the range of uncertainty suggested by our methods.

Finally, some national censuses collect data on deaths within households. As with vital registration data, censuses have varying levels of completeness, and like sibling history data, household deaths reported in censuses may be underreported [37]. Figures 8–10 show that census-based mortality rates in Zimbabwe, Malawi, and Tanzania are remarkably close to the CSS estimates at various time periods, whereas in Mauritania (Figure 7), census data yield dramatically lower levels.

While this is not necessarily validation, the fact that CSS yields estimates that are comparable to those from independent data collection schemes is reassuring.

### Mortality Trends

Figures 5–10 and S2 also show how risks of adult death have changed over the period 1980–2005, encompassing the peak effects of the HIV/AIDS epidemic, particularly in Africa. In some countries, notably Cote d'Ivoire, Cameroon, Kenya, Lesotho, Malawi, and Swaziland, death rates among adults appear to have risen throughout the past two decades or so. In Malawi and Zimbabwe, they have increased 3- to 4-fold since the late 1980s, with CSS showing the full devastation of the epidemic on adult survival. In Kenya, Zambia, Swaziland, and Tanzania, death rates have doubled in 20 y, although in Tanzania and Zambia at least, there are signs that death rates may be stabilizing. In others, particularly Congo, Ethiopia, and Madagascar, we have identified increases in mortality followed by declines. The effect of the 1994 genocide in Rwanda can be clearly seen, after which death rates dropped to levels similar to neighboring African countries. Haiti, Morocco, and Peru have experienced consistent declines in adult mortality over the past two decades. One important consideration in examining these trend results is that in adjusting for recall bias, we are using a fixed estimate of recall bias for all of these countries. As we have shown, there are likely some differences across countries in the magnitude of recall bias. Imposing a fixed correction for a country that is not like the average countries in our dataset will result in distortions of the real trend. Nevertheless, as we see from Figures 2 and 3, the change in the trend that the recall bias correction induces is small compared to some of the trends seen in the data such as those in Zimbabwe, Uganda, and Tanzania. Less clear is whether the slight short-term fluctuations seen in countries like Bolivia, Senegal, and Indonesia are real. While it may be difficult to interpret short-term changes in death rates, except for the clear effect of the genocide in Rwanda, the utility of the method in determining longer term trends in mortality levels is clearly of great public health importance.

**Figure 11:**
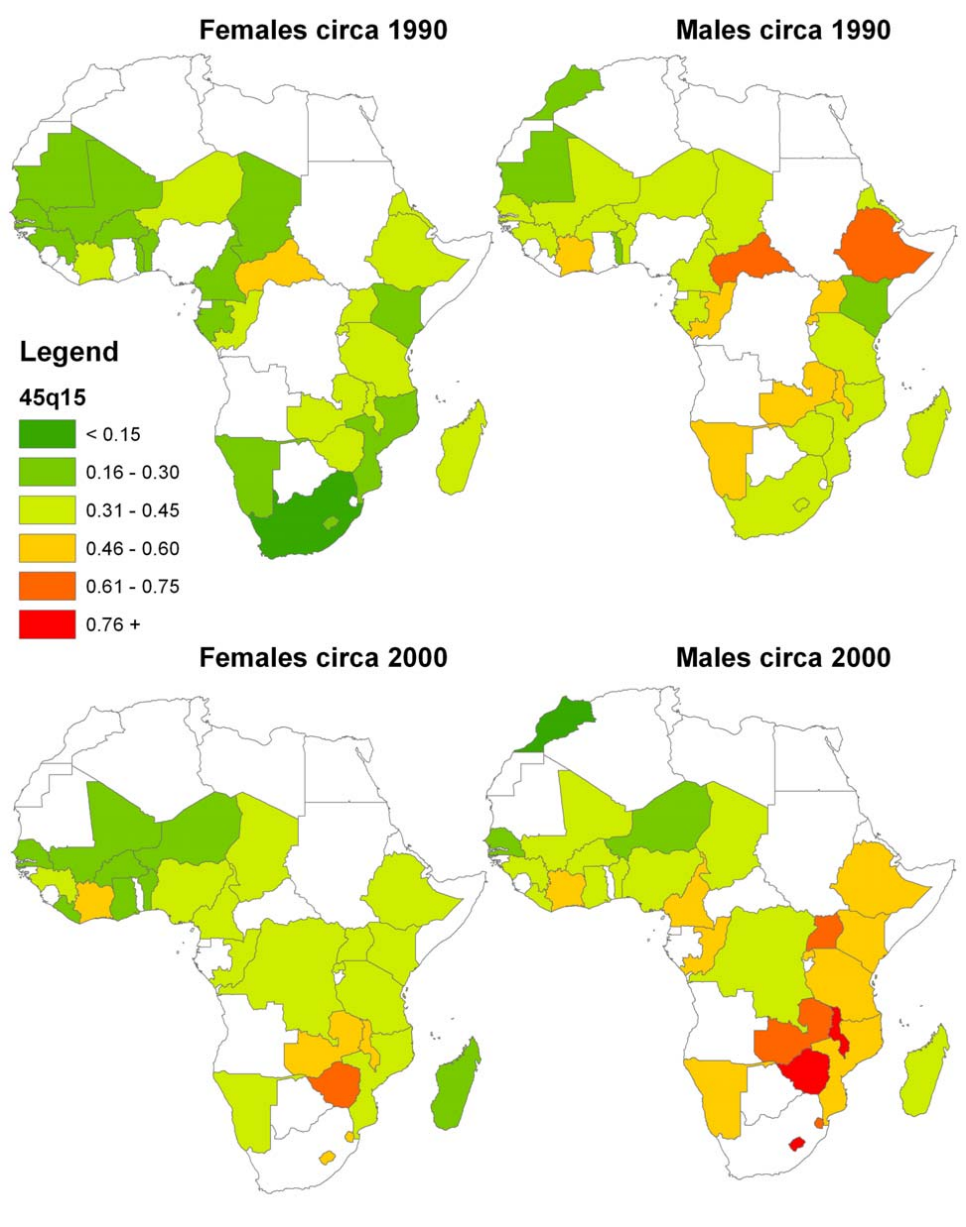
CSS-generated estimates of _45_ *q*_15_ for African countries with DHS data, 1990 and 2000.

A summary appraisal of trends in adult mortality in Africa can be obtained from Figure 11, which shows levels of mortality estimated for various countries around 1990 (1988–1992) and 2000 (1998–2002). For women, the dramatic rise in adult mortality in Zambia is clear, as are the relatively low levels of mortality prevailing around 1990 in countries such as Morocco, Senegal, Benin, Kenya, and South Africa. The greater heterogeneity of mortality levels around 2000 can also be seen, largely due to the differential impact of the HIV epidemic. For men, the heterogeneity among countries is greater than for women, even in 1990, potentially reflecting the greater risks they incur from injuries and violence. The impact of the HIV epidemic is also clear from Figure 11, particularly in the Southern African states around 2000.

## Discussion

In this paper, we presented an improved method for analyzing sibling survival data that adjusts for two key forms of bias present in survey data: survival and recall bias. We demonstrated its application using 83 surveys undertaken in 44 countries. This method greatly expands our direct empirical knowledge of levels and trends in adult mortality in developing countries without resorting to the use of demographic model life tables. Adult mortality measurement from empirical data will decrease the dependence of the global health community on uncertain predictions from levels of child mortality and provide for better tracking of progress toward major health and development targets.

Collective concerns about the low levels of adult mortality from crude analysis of sibling data [1],[15] may have dampened enthusiasm for collecting this type of data in the global health community. We believe that the CSS method provides grounds for renewed optimism in collecting sibling survival data. Our experience argues for all DHS surveys and similarly sized national health surveys to incorporate the sibling history module, as even in middle-income countries this information could be a useful adjunct to analyzing levels and trends in adult mortality from vital registration data, especially where it may only be partially complete. Widespread collection of these data will greatly strengthen our capacity to monitor maternal mortality and the ultimate effect of interventions such as antiretrovirals in reducing adult mortality.

In addition to expanding the collection of sibling survival data in more surveys, our analysis suggests that the set of respondents who answer the sibling module in a survey should be expanded. By only asking women aged 15–49 y, the current DHS practice limits our ability to effectively measure mortality in adults over age 50 y and for older time periods, especially for more than 15 y prior to the survey. To achieve sufficient numbers at these older ages, we have pooled the data across countries and assumed four constant age patterns. An expanded age range of respondents would allow for the relaxation of this assumption and estimation of more specific and stable age patterns. As it is, the precise age patterns of mortality generated from this empirical model, especially above age 50, may not be accurate. Further, if both male and female respondents were to be asked the sibling history module, exploration of sex-specific biases in the recall of births and deaths of sisters and brothers would be possible and would allow for cross-validation. There is some evidence from our analysis that this effect may be significant. With increased concerns about the early impact of the epidemiological transition in many developing countries, expanding the age range of respondents will allow direct measurement of middle-aged mortality in these countries. Given that survey teams in the DHS and other survey programs are already visiting households, expanding the set of respondents who are asked the sibling history module would not imply a substantial marginal cost on survey implementers. Our experience suggests that the information obtained is likely to be well worth the investment.

A key limitation of our analysis is the estimation of the average recall bias across all surveys and the use of this average effect in calculating levels and trends in _45_ *q*_15_. Six of 18 country- and sex-specific estimates of recall bias are statistically significantly different from the average estimate of recall bias. The differences could affect estimates of trends for these countries and others for which fewer than three surveys are available. In the absence of sufficient overlapping surveys in a country, using the average recall bias estimate is necessary. As more countries accumulate multiple surveys, it will become possible to apply the CSS model on a regional and eventually even country-by-country basis. Country-specific recall bias parameter values can then be used in generating country-specific levels of adult mortality. As more countries collect sibling survival data, it will also be possible to explore the contextual, linguistic, and other cultural factors that might account for variability in recall bias. This type of insight should help to guide further improvements in survey instruments for sibling recall. Experiments are underway to explore alternative wording of the sibling history module in Tanzania, India, and the Philippines as part of the Gates Grand Challenges in Global Health initiative [38]. Growing recognition of the potential utility of sibling history data for public health monitoring will hopefully stimulate more research in this area.

The prospect that robust information on the levels and trends in adult mortality can be derived from periodic household surveys in low-income countries may warrant a reconsideration of the priorities for improved assessment of adult mortality. The MOVE group [39] called for an expansion of vital registration systems and the use of sample registration systems in the interim. Those are important initiatives, but our findings suggest that it may be as important to more persuasively argue for the inclusion of sibling survival modules in ongoing survey programs. Further work is also needed to explore the feasibility of using new verbal autopsy instruments and analytical methods in conjunction with these modules to ascertain not only death rates, but also causes of death [40],[41]. The demand for accountability and the use of pay for performance investments such as the Global Alliance for Vaccines and Immunisation (GAVI) is likely to increase the pressure on countries to mount more frequent household surveys [42]. Maximum use of these opportunities should be made for tracking trends in adult mortality.

These opportunities must be seized if we are to more reliably understand the levels, patterns, and causes of adult mortality and how they are changing. Parallel investments in vital registration systems and the routine inclusion of sibling survival questions into existing or planned household survey programs are urgently needed if we are to rapidly build the evidence base for public health action. The success of child survival programs, accompanied by greater global concern for controlling major threats to health, argue for much greater research attention to be given to measuring adult mortality and its causes. Keeping children alive to adulthood is a noble and worthy aim for the global public health community; keeping young adults alive and in good health until they reach old age should be seen as equally important.

## Supporting Information

Figure S1 The relationship between percent dead and sibship size for males and females, using an example from the Mali 2006 DHS. The percent dead for females has been corrected in sibship sizes 1, 2, and 3 to account for the increased occurrence of zero-female-survivors in these sibships.
(0.38 MB TIF)

Figure S2 Estimates of _45_q_15_ from the CSS method for all 44 countries where sibling history surveys are available in the DHS.
(7.22 MB PDF)

Table S1 Characteristics of the sibling history surveys in 83 DHS.
(0.04 MB XLS)

Table S2 CSS age-pattern sensitivity analysis: Parameter results from models 1–4.
(0.23 MB XLS)

Table S3 CSS age-pattern sensitivity analysis: Estimated _45_q_15_ and 95% uncertainty intervals for all country-periods, models 1–4.
(0.23 MB XLS)

Text S1 Derivation of zero-female-survivor correction.
(0.03 MB PDF)

## Abbreviations

CSS: Corrected Sibling Survival
DHS: Demographic and Health Surveys
DSS: Demographic Surveillance Site
GK: Gakidou and King
RMSE: root mean squared error
*TiPS*: time prior to the survey
