# Supplementary figures and images for: Measuring Adult Mortality Using Sibling Survival: A New Analytical Method and New Results for 44 Countries, 1974–2006
Source: PLoS Med. 2010 Apr 13;7(4):e1000260. doi: 10.1371/journal.pmed.1000260 (PMC2854132; doi:10.1371/journal.pmed.1000260)

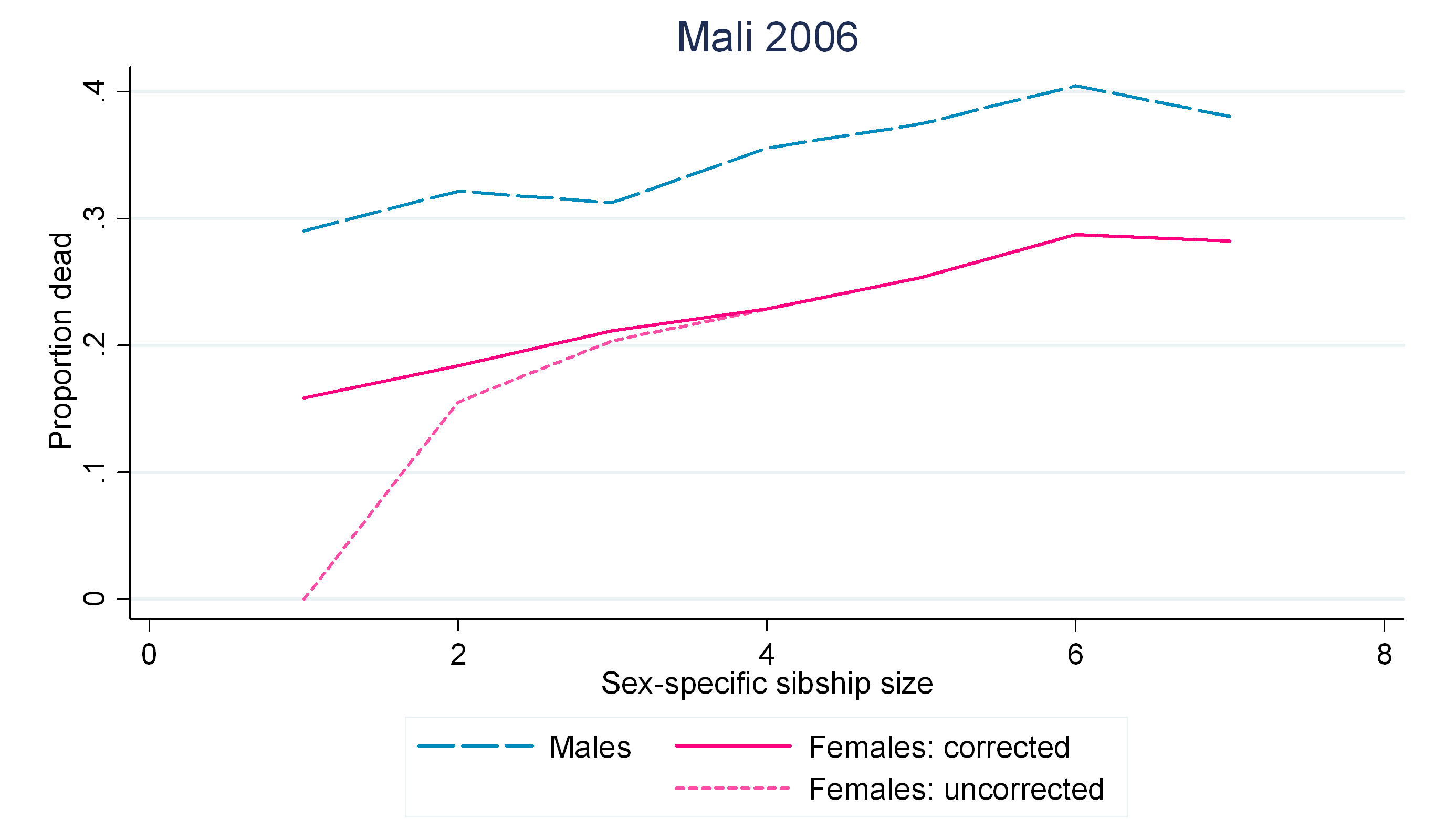

Supplement: Figure S1 — The relationship between percent dead and sibship size for males and females, using an example from the Mali 2006 DHS. The percent dead for females has been corrected in sibship sizes 1, 2, and 3 to account for the increased occurrence of zero-female-survivors in these sibships. (0.38 MB TIF) [file pmed.1000260.s001.tif]
