# Supplementary material for: Measuring Adult Mortality Using Sibling Survival: A New Analytical Method and New Results for 44 Countries, 1974–2006
Source: PLoS Med. 2010 Apr 13;7(4):e1000260. doi: 10.1371/journal.pmed.1000260 (PMC2854132; doi:10.1371/journal.pmed.1000260)
Supplement: Figure S2 — Estimates of 45q15 from the CSS method for all 44 countries where sibling history surveys are available in the DHS. (7.22 MB PDF) [file pmed.1000260.s002.pdf]

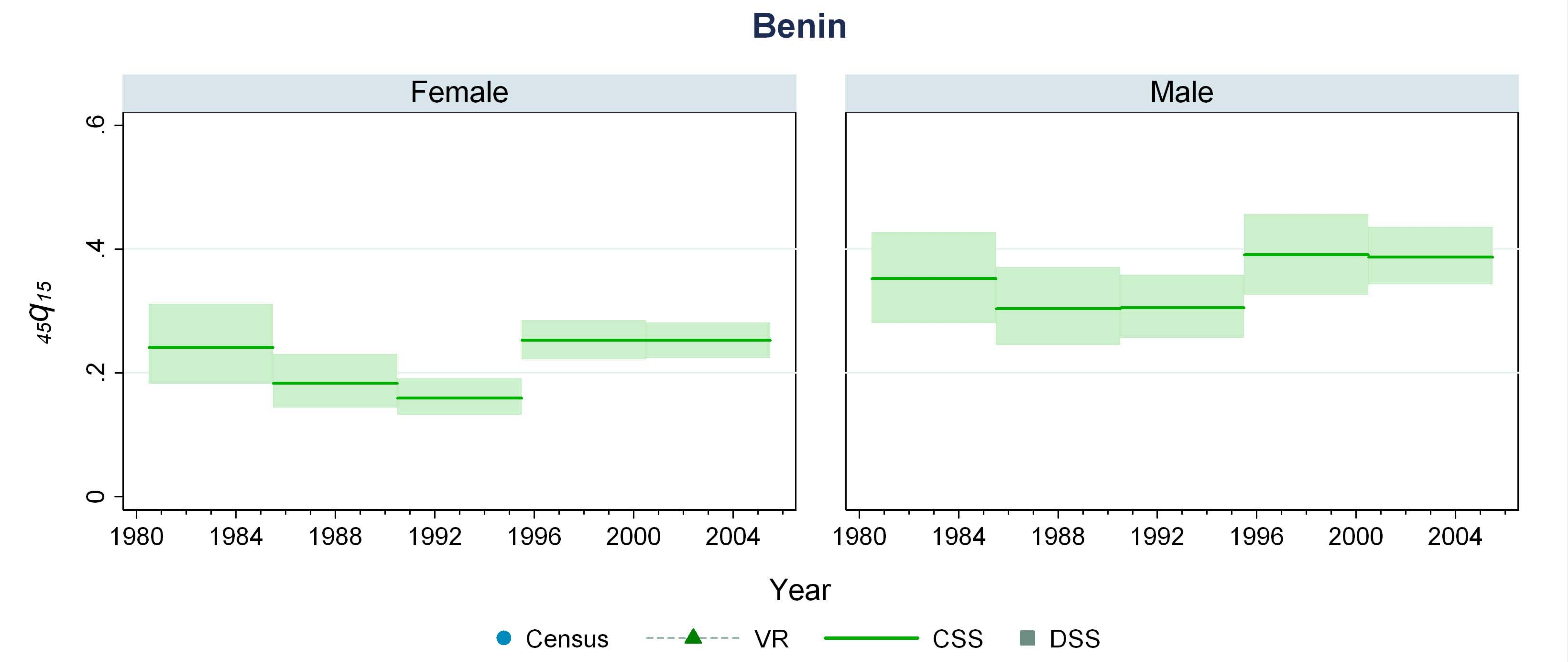
Benin
Female
Male
$_{45}q_{15}$
0
.2
.4
.6
1980
1984
1988
1992
1996
2000
2004
Year
Census
VR
CSS
DSS

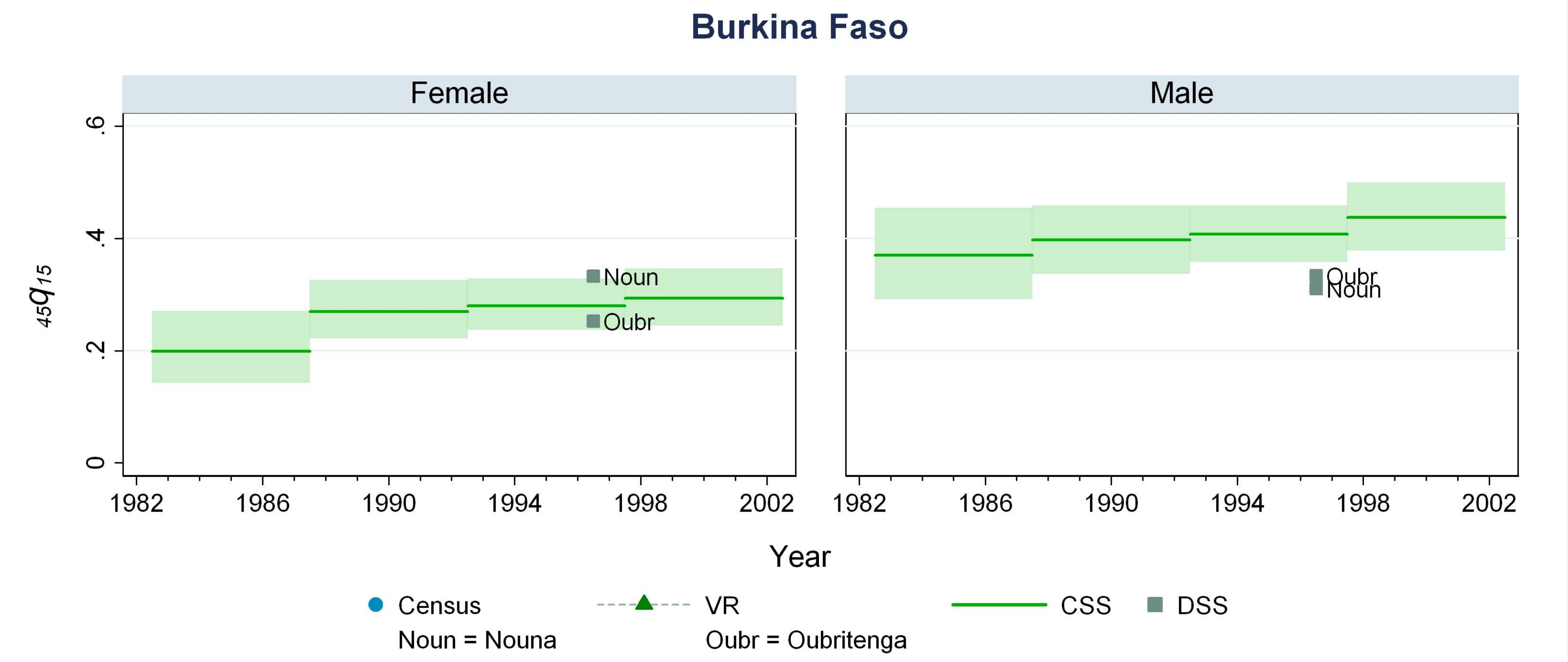
Burkina Faso
Female
Male
$_{45}q_{15}$
0
.2
.4
.6
1982
1986
1990
1994
1998
2002
Noun
Oubr
Year
Census
VR
CSS
DSS
Noun = Nouna
Oubr = Oubritenga

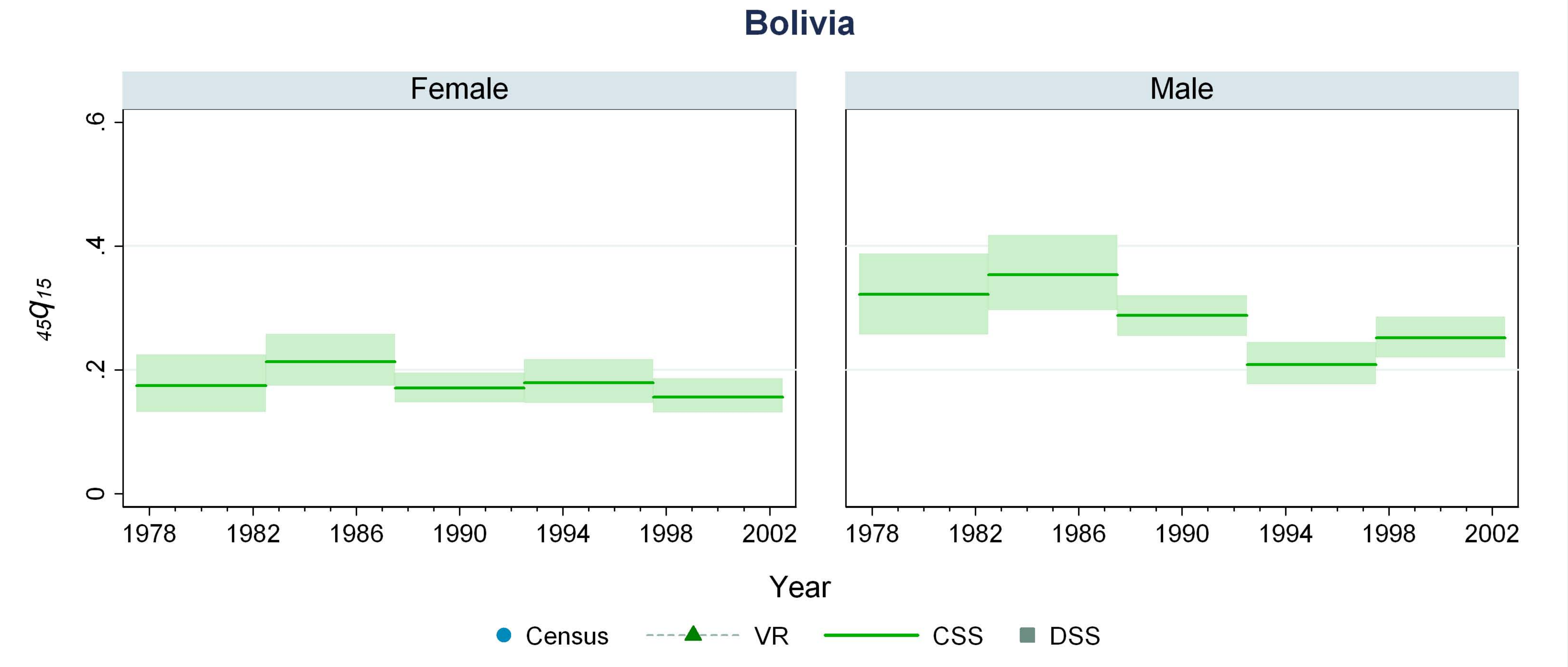
Bolivia
Female
Male
$_{45}q_{15}$
0
.2
.4
.6
1978
1982
1986
1990
1994
1998
2002
Year
Census
VR
CSS
DSS

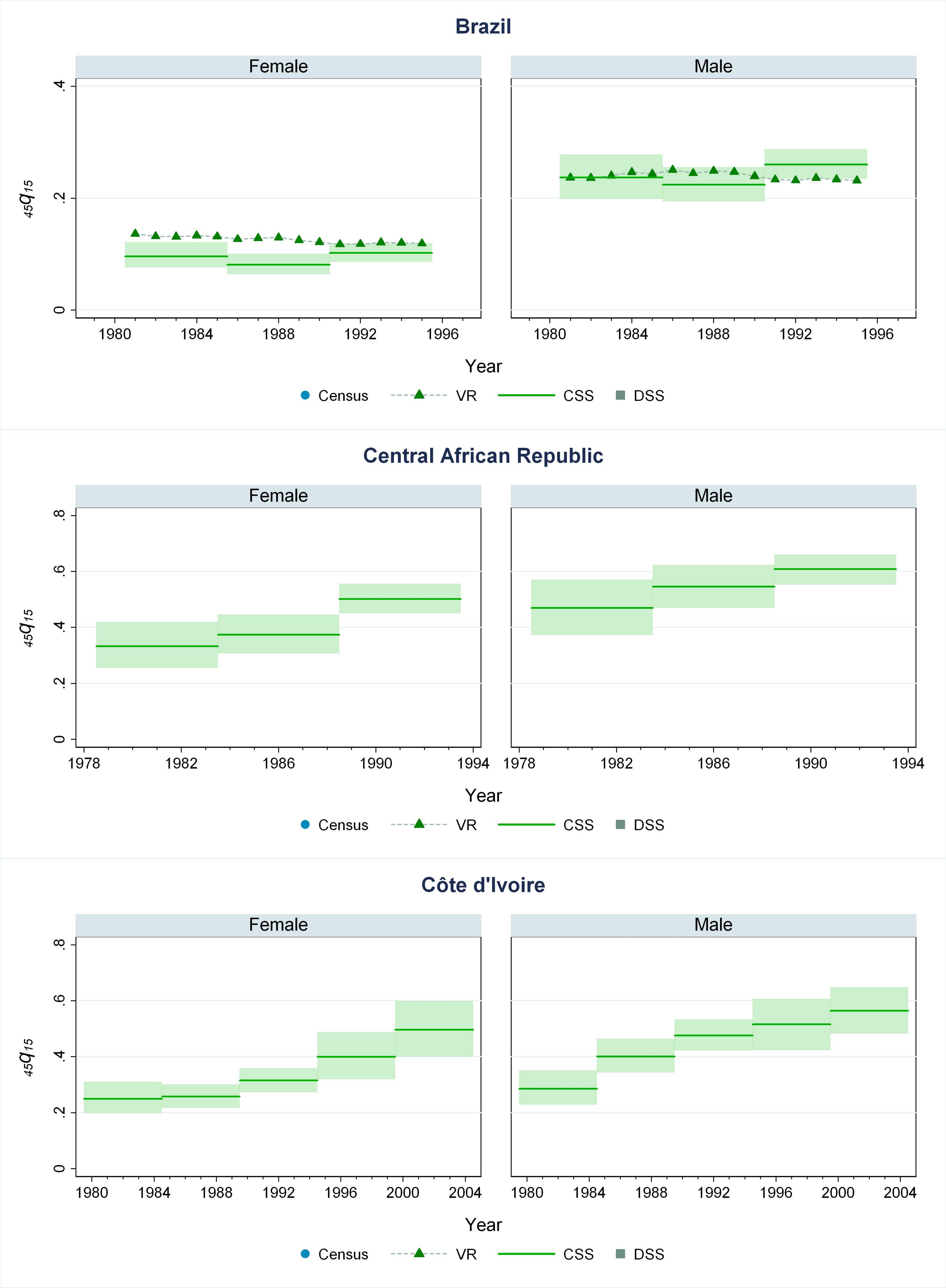

Brazil
Female
Male
$_{45}q_{15}$
0
.2
.4
1980
1984
1988
1992
1996
Year
Census
VR
CSS
DSS
Central African Republic
Female
Male
$_{45}q_{15}$
0
.2
.4
.6
.8
1978
1982
1986
1990
1994
Year
Census
VR
CSS
DSS
Côte d'Ivoire
Female
Male
$_{45}q_{15}$
0
.2
.4
.6
.8
1980
1984
1988
1992
1996
2000
2004
Year
Census
VR
CSS
DSS

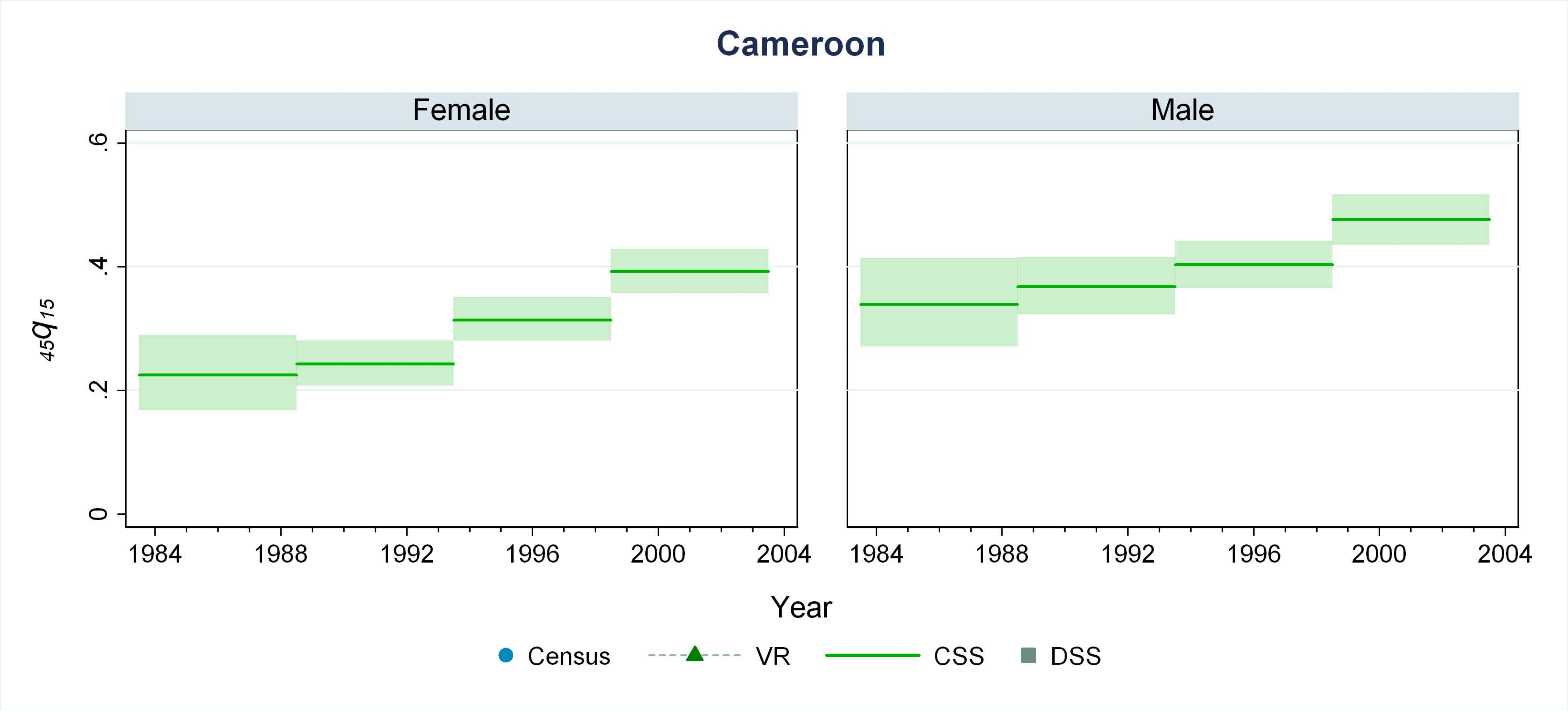
Cameroon
Female
Male
$_{45}q_{15}$
0
.2
.4
.6
1984
1988
1992
1996
2000
2004
Year
Census
VR
CSS
DSS

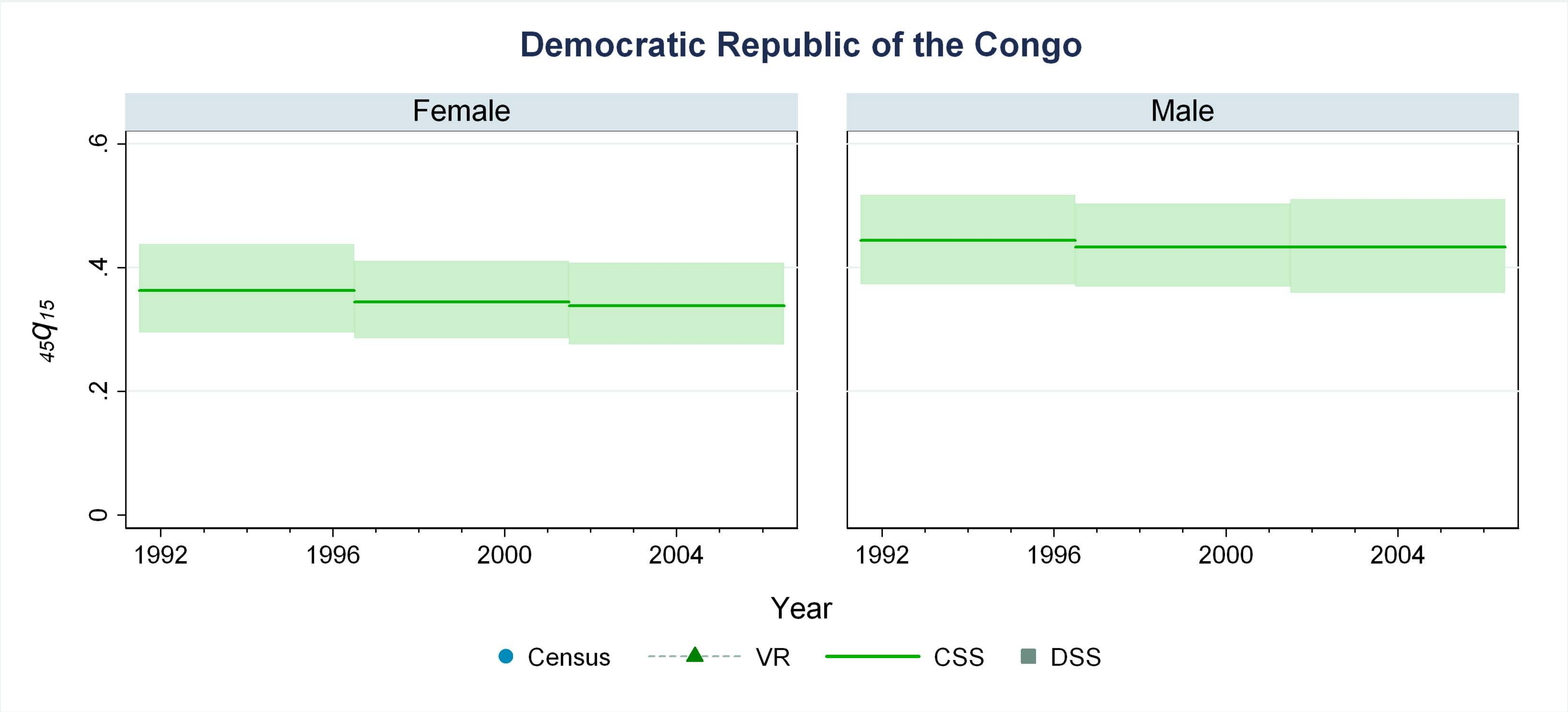
Democratic Republic of the Congo
Female
Male
$_{45}q_{15}$
0
.2
.4
.6
1992
1996
2000
2004
Year
Census
VR
CSS
DSS

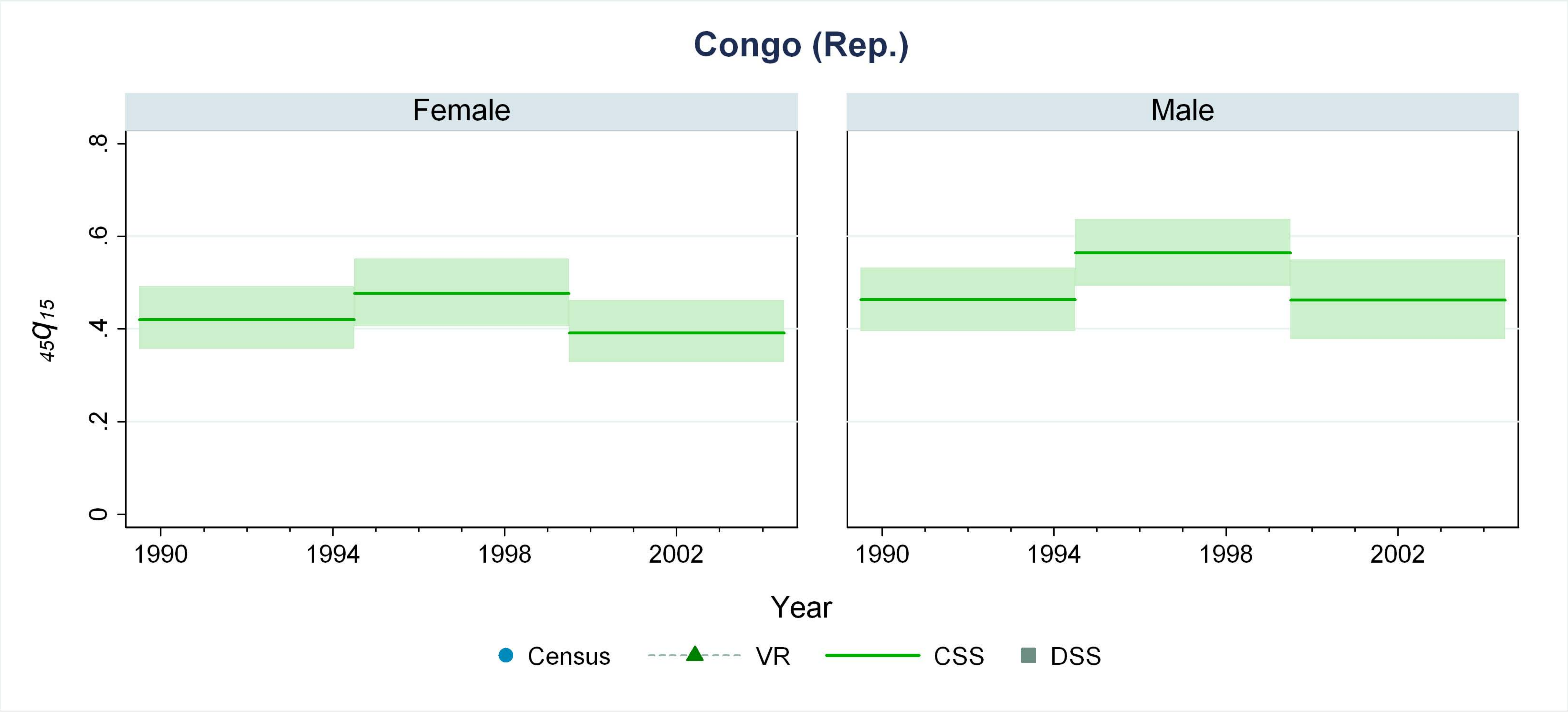
Congo (Rep.)
Female
Male
$_{45}q_{15}$
0
.2
.4
.6
.8
1990
1994
1998
2002
Year
Census
VR
CSS
DSS

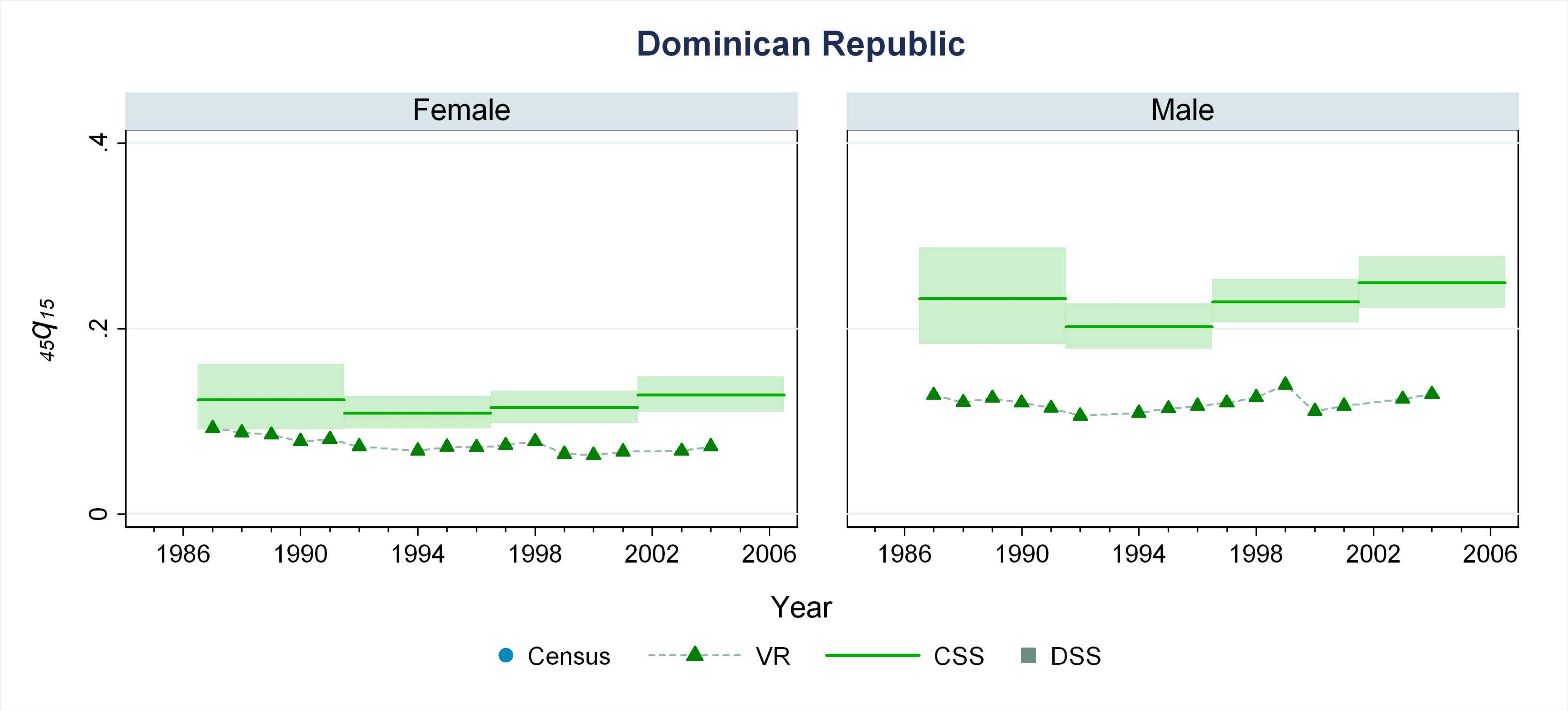
Dominican Republic
Female
Male
$_{45}q_{15}$
0
.2
.4
1986
1990
1994
1998
2002
2006
Year
Census
VR
CSS
DSS

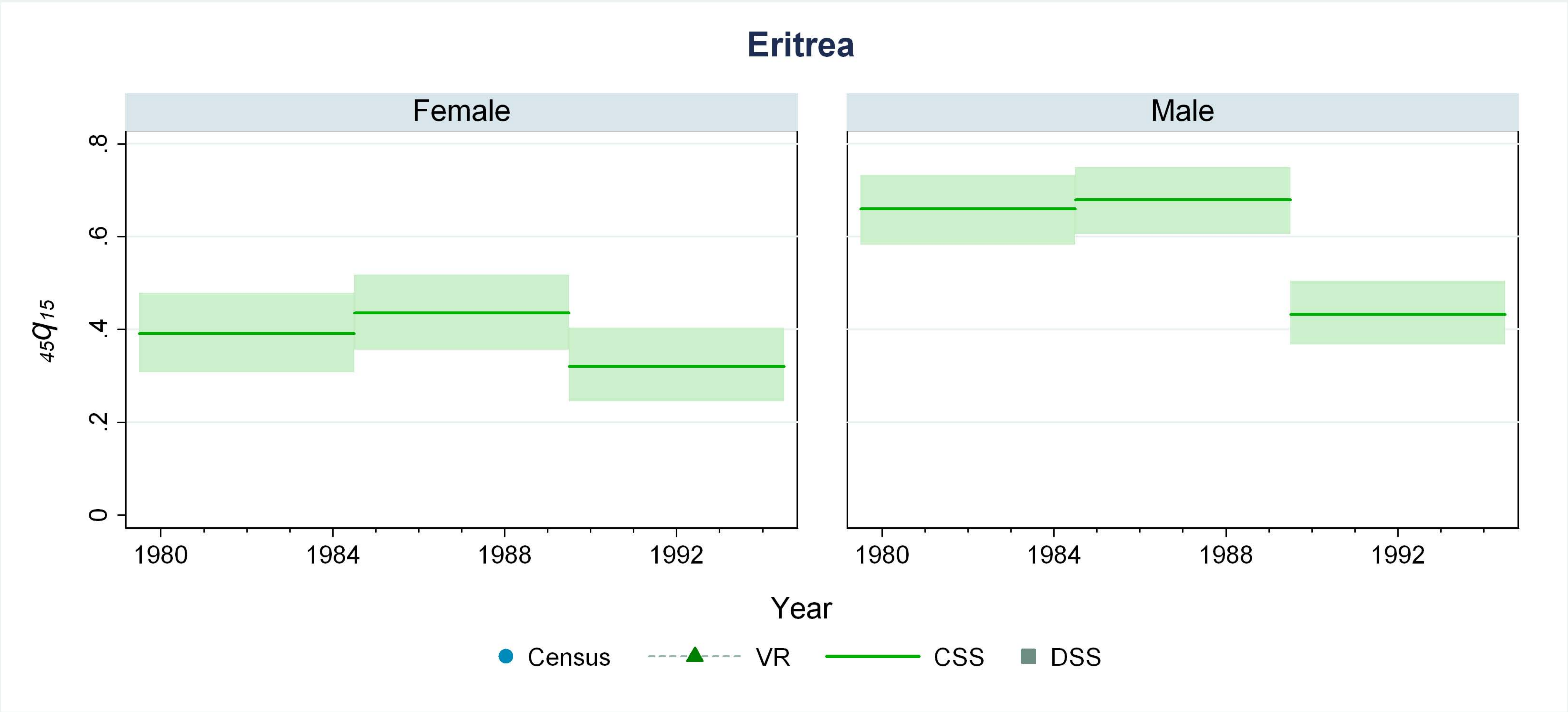
Eritrea
Female
Male
$_{45}q_{15}$
0
.2
.4
.6
.8
1980
1984
1988
1992
Year
Census
VR
CSS
DSS

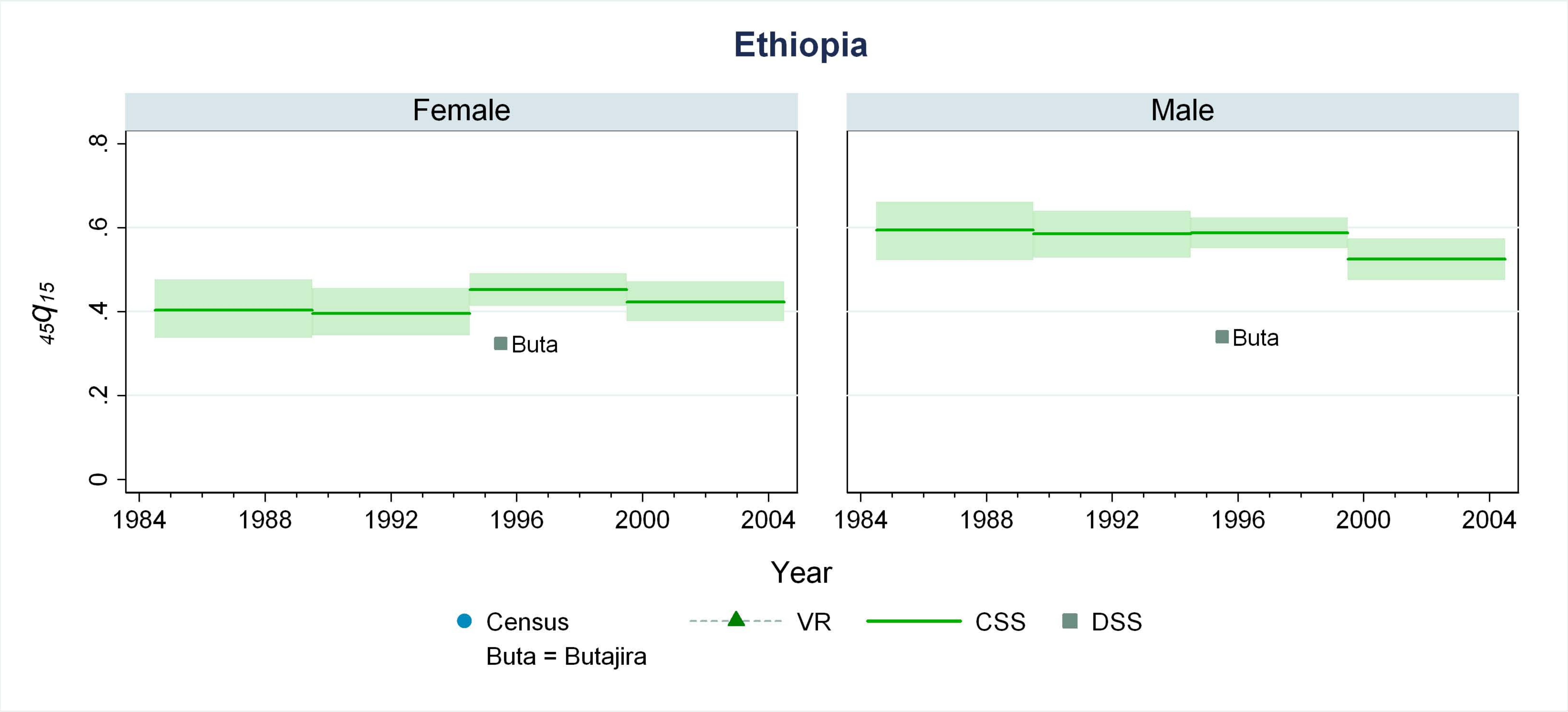
Ethiopia
Female
Male
$_{45}q_{15}$
0
.2
.4
.6
.8
Buta
1984
1988
1992
1996
2000
2004
Year
Census
VR
CSS
DSS
Buta = Butajira

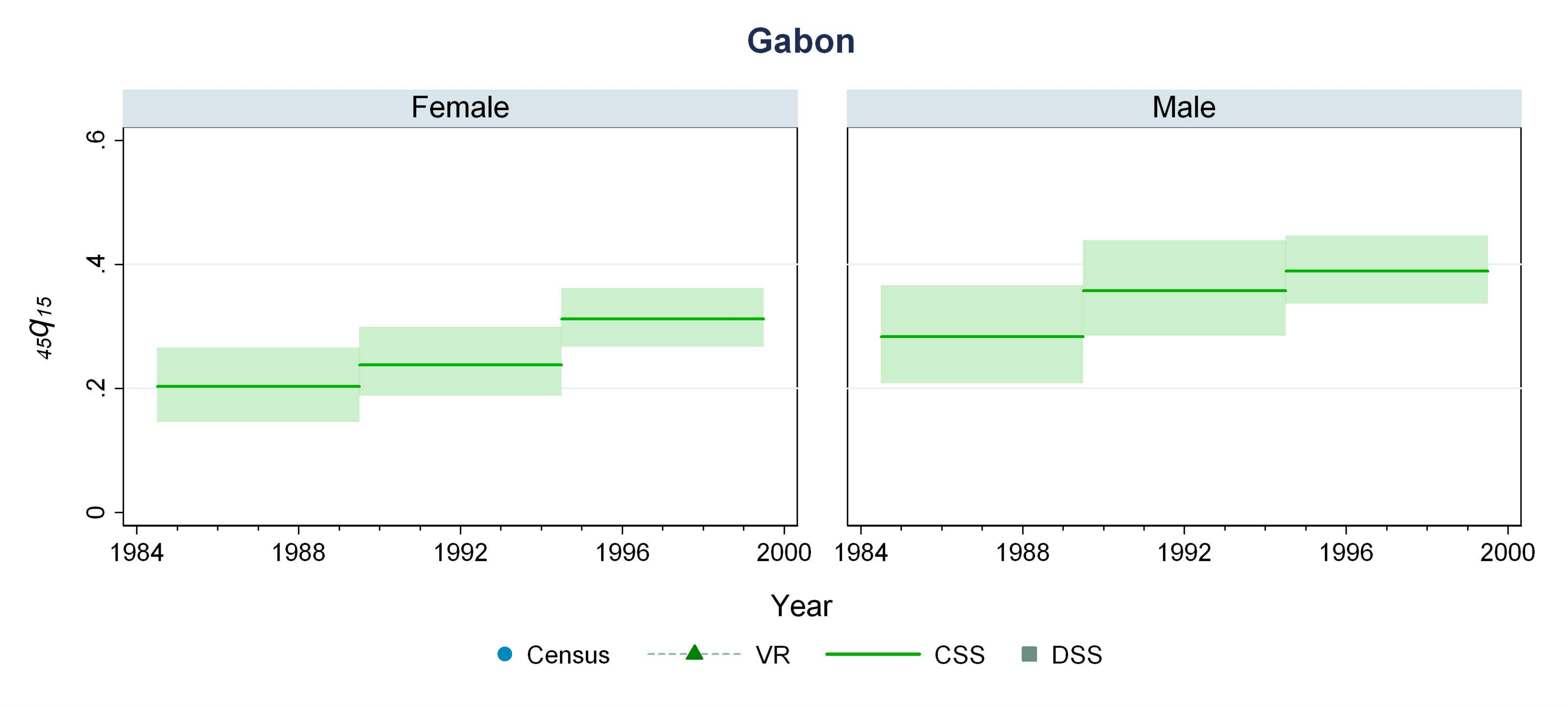
Gabon
Female
Male
$_{45}q_{15}$
0
.2
.4
.6
1984
1988
1992
1996
2000
Year
Census
VR
CSS
DSS

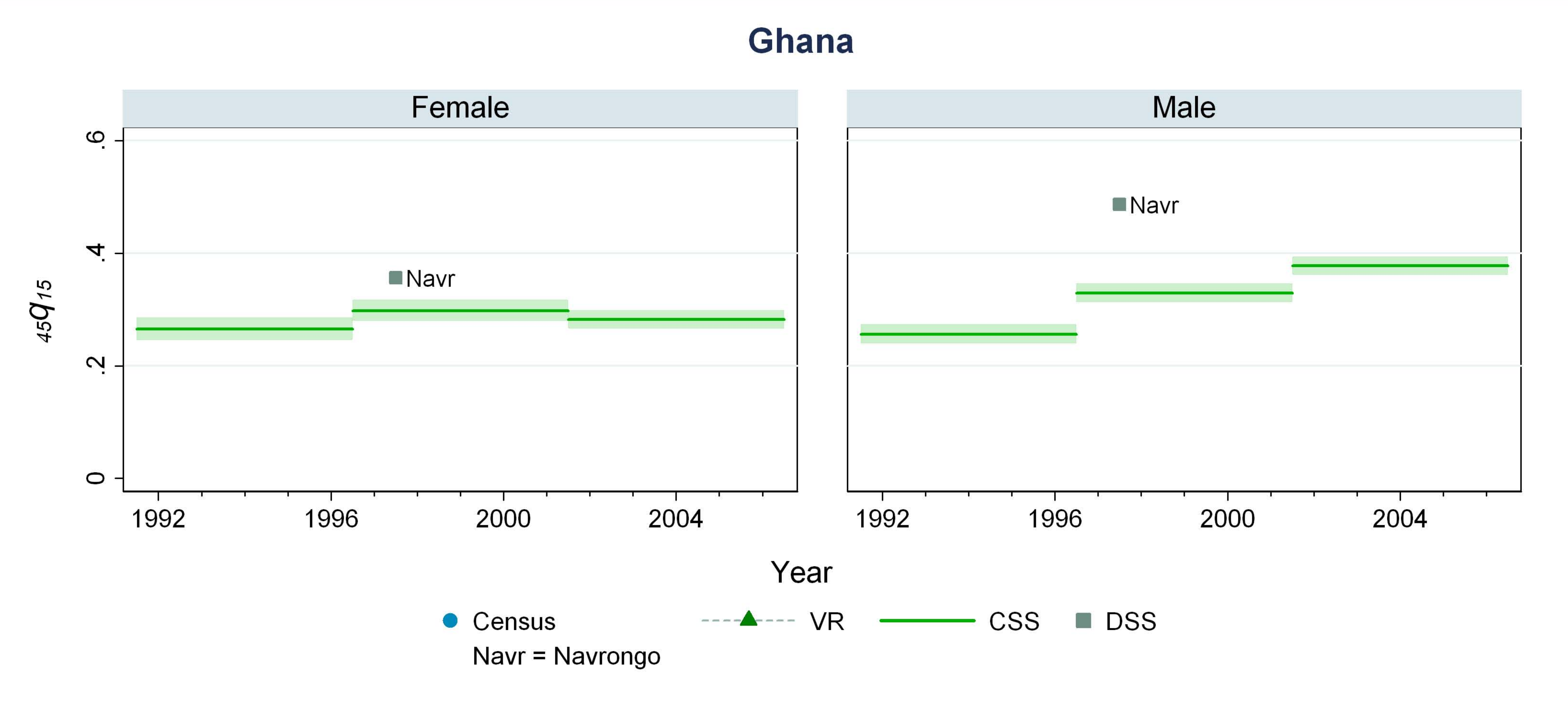
Ghana
Female
Male
$_{45}q_{15}$
0
.2
.4
.6
Navr
1992
1996
2000
2004
Year
Census
VR
CSS
DSS
Navr = Navrongo

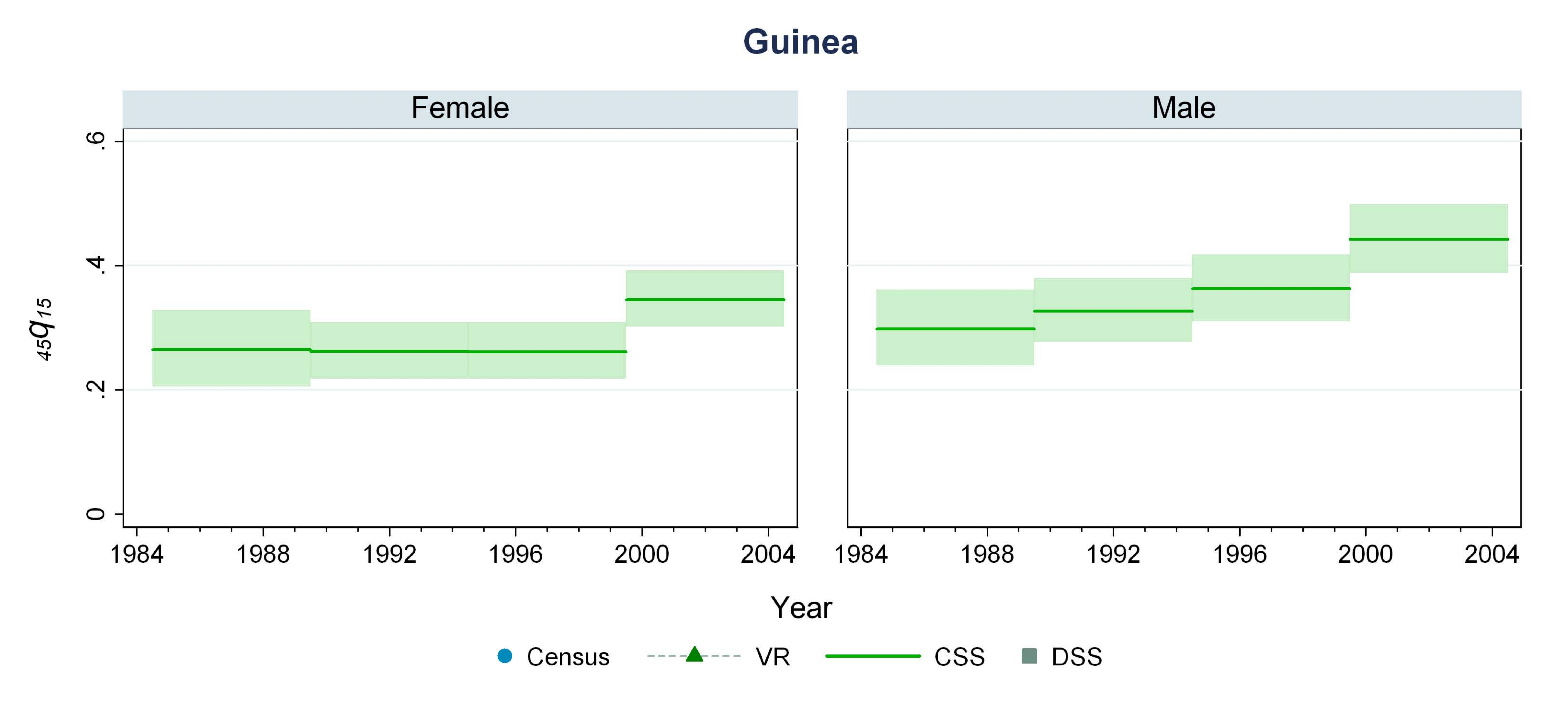
Guinea
Female
Male
$_{45}q_{15}$
0
.2
.4
.6
1984
1988
1992
1996
2000
2004
Year
Census
VR
CSS
DSS

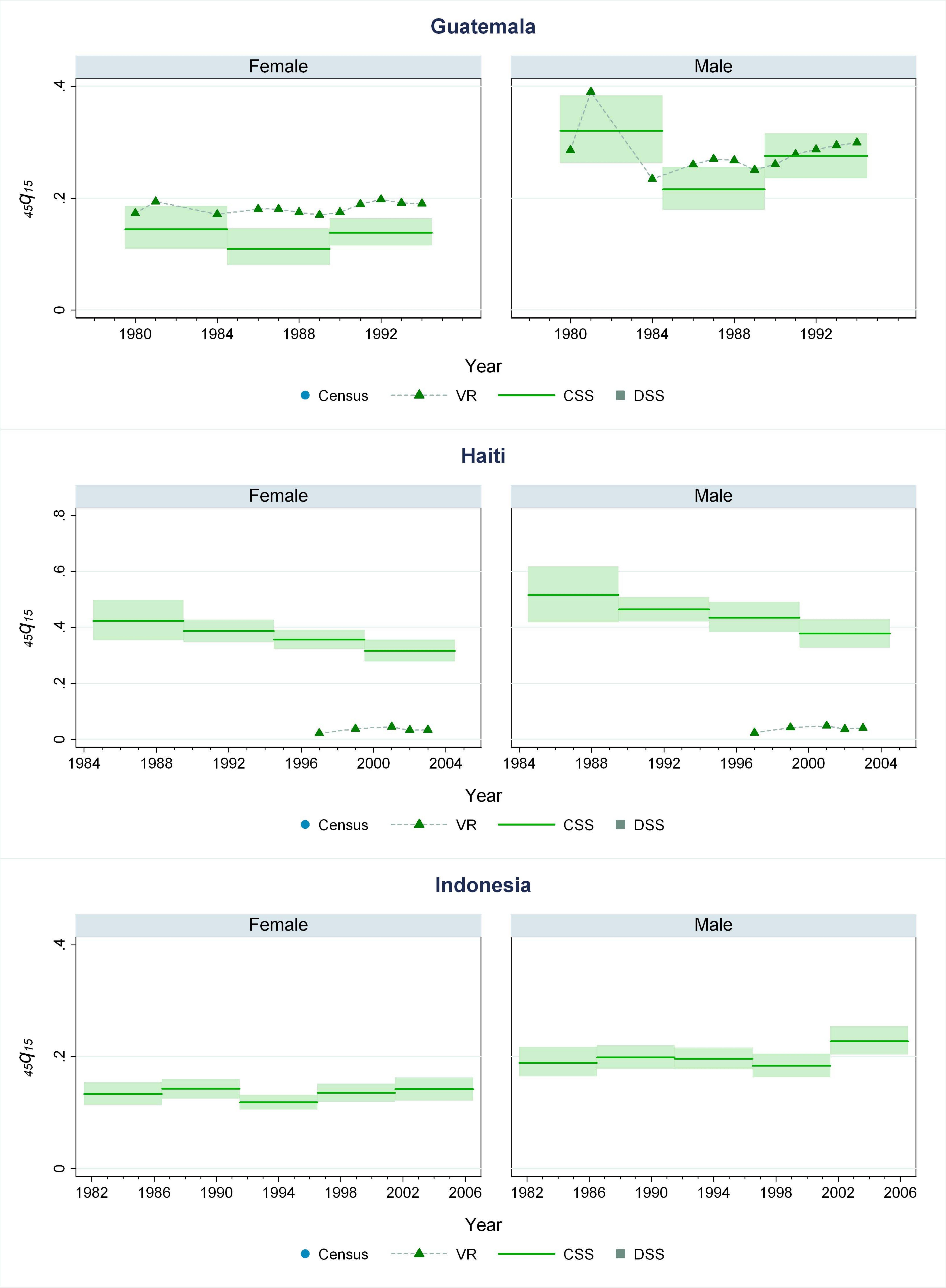
Guatemala
Female
Male
$_{45}q_{15}$
0
.2
.4
1980
1984
1988
1992
Year
Census
VR
CSS
DSS
Haiti
Female
Male
$_{45}q_{15}$
0
.2
.4
.6
.8
1984
1988
1992
1996
2000
2004
Year
Census
VR
CSS
DSS
Indonesia
Female
Male
$_{45}q_{15}$
0
.2
.4
1982
1986
1990
1994
1998
2002
2006
Year
Census
VR
CSS
DSS

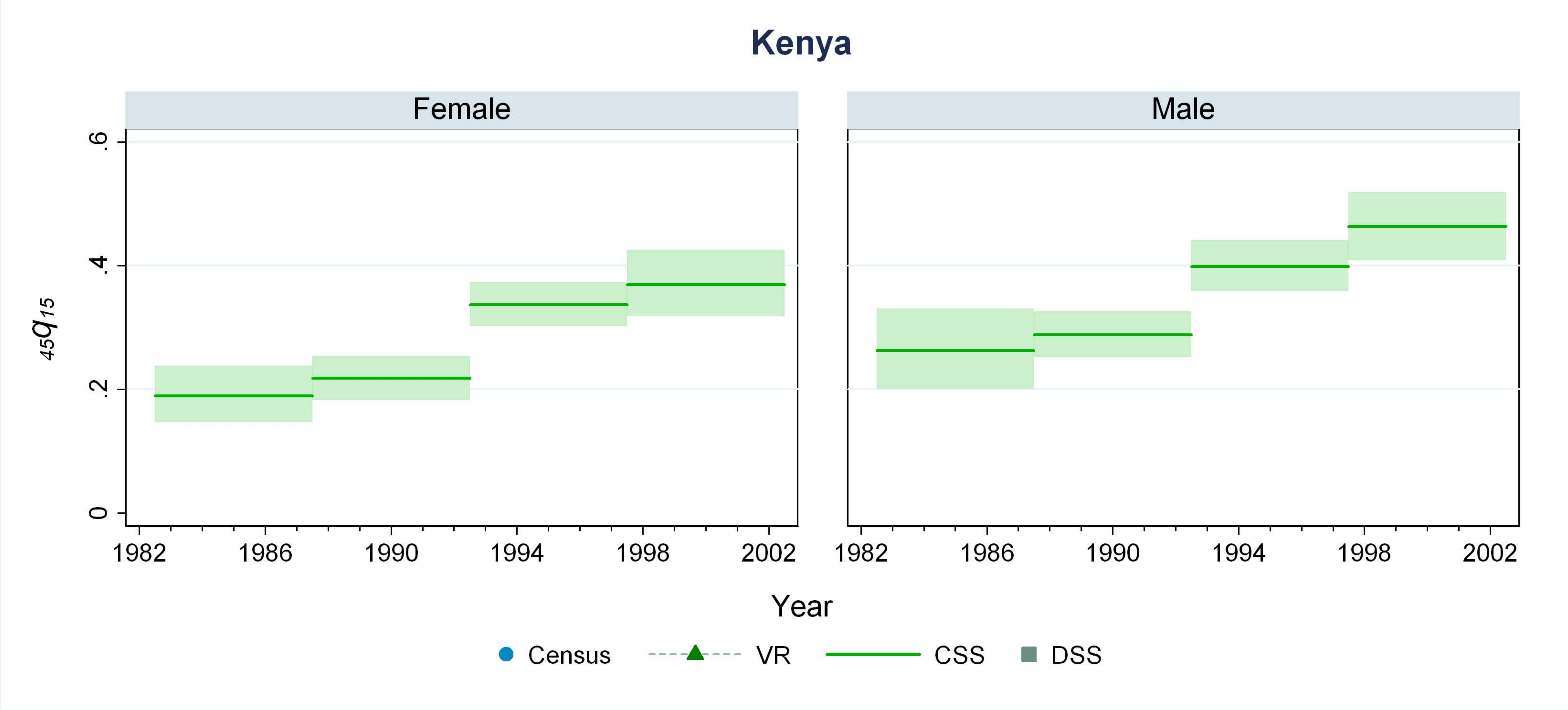
Kenya
Female
Male
0
.2
.4
.6
45q15
1982
1986
1990
1994
1998
2002
Year
Census
VR
CSS
DSS

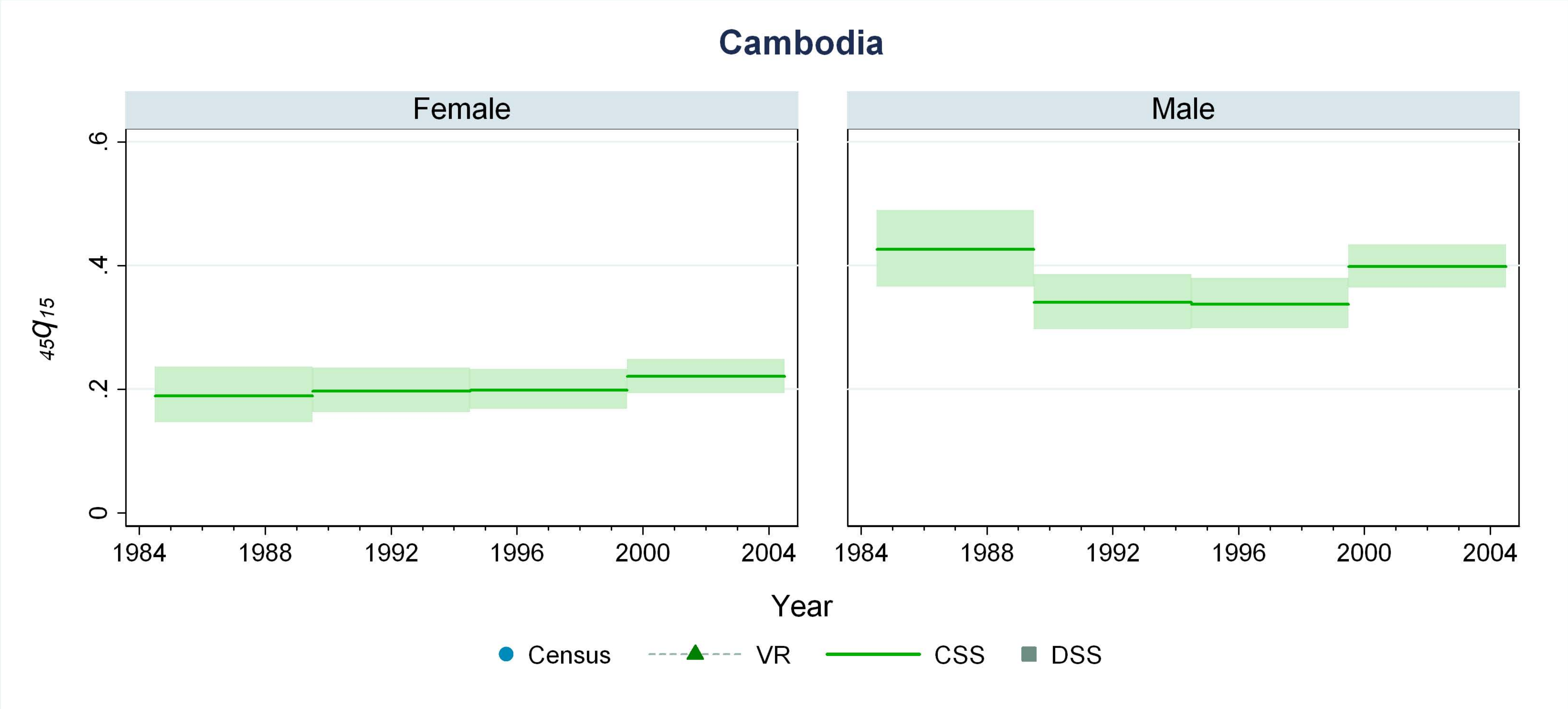
Cambodia
Female
Male
0
.2
.4
.6
45q15
1984
1988
1992
1996
2000
2004
Year
Census
VR
CSS
DSS

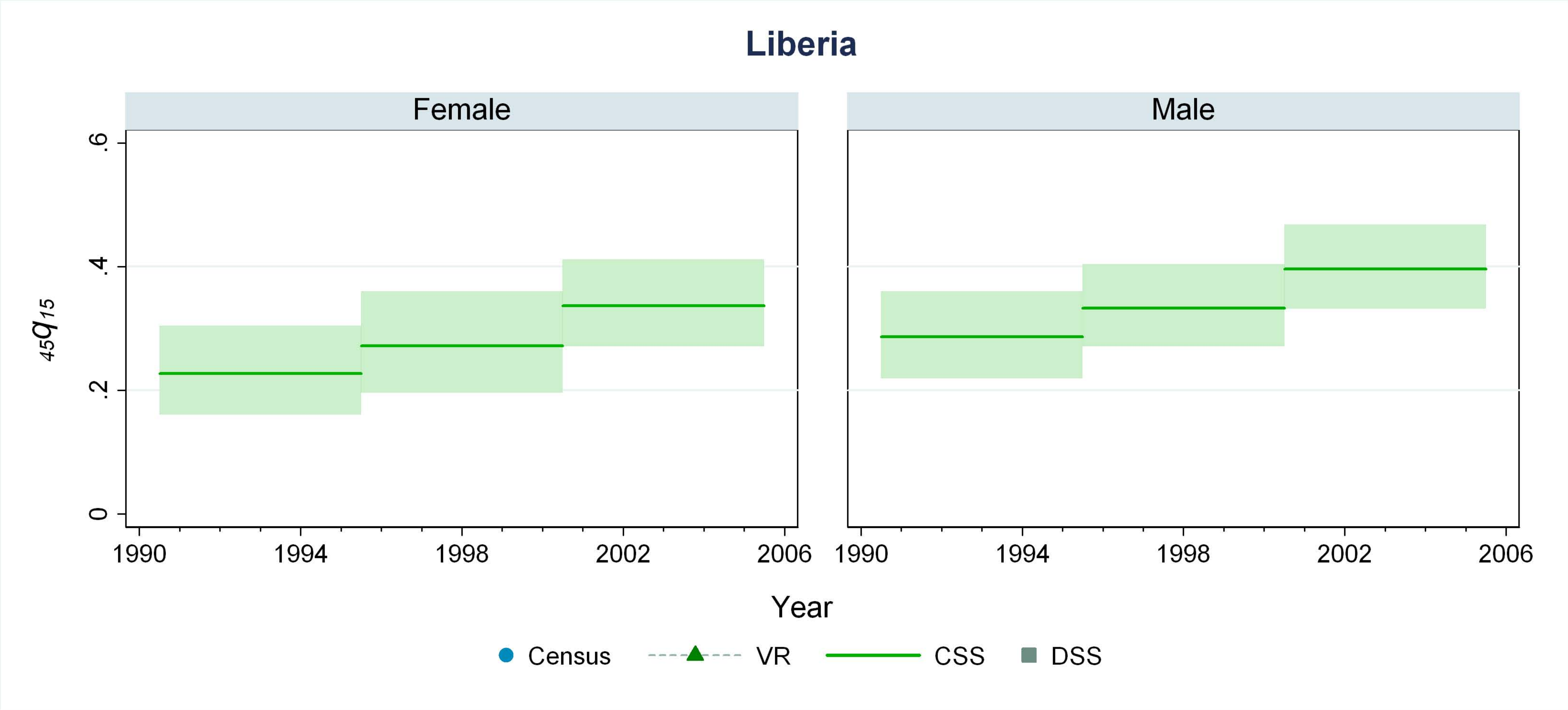
Liberia
Female
Male
0
.2
.4
.6
45q15
1990
1994
1998
2002
2006
Year
Census
VR
CSS
DSS

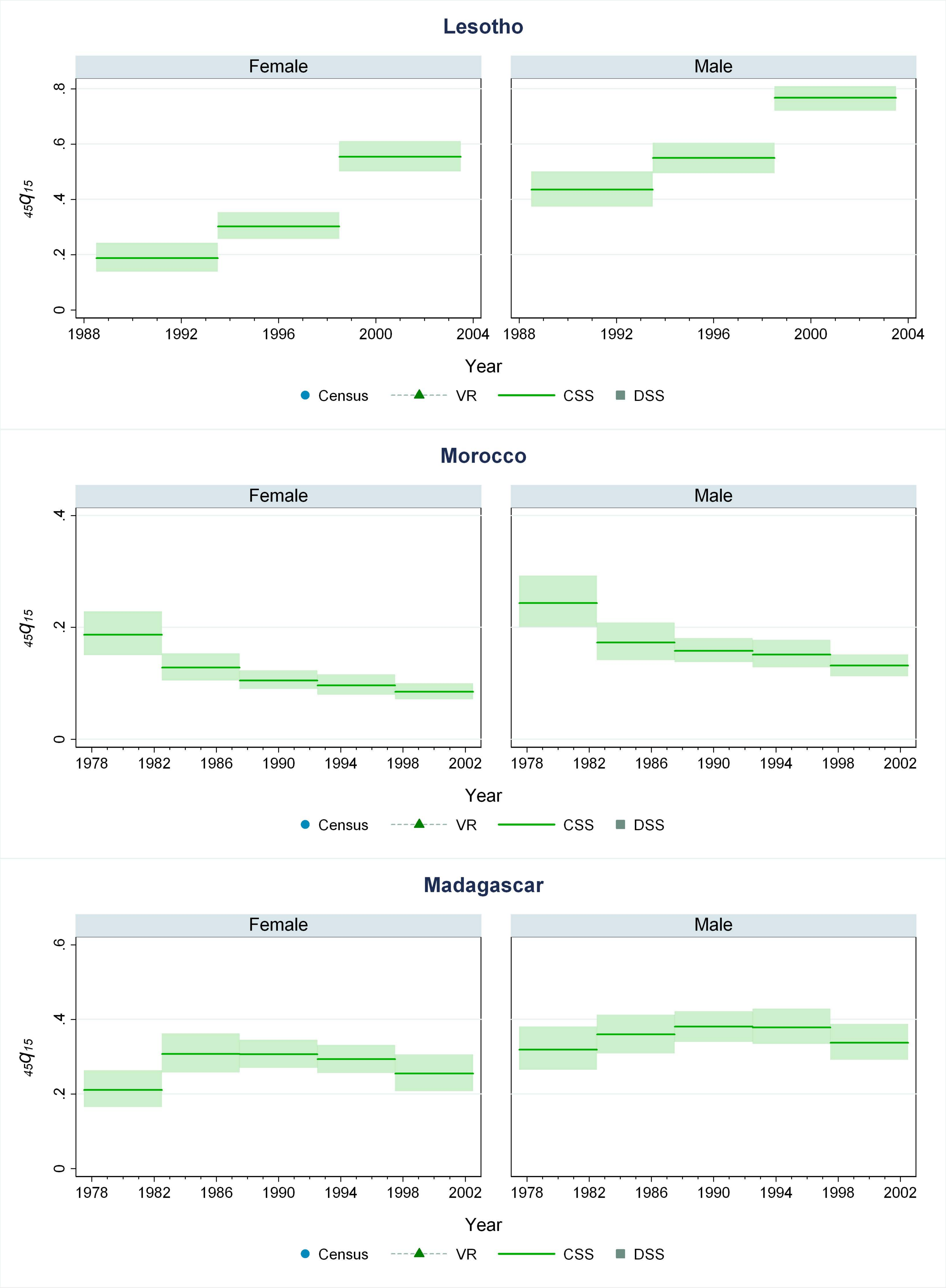

Lesotho
Female
Male
$_{45}q_{15}$
0
.2
.4
.6
.8
1988
1992
1996
2000
2004
Year
Census
VR
CSS
DSS
Morocco
Female
Male
$_{45}q_{15}$
0
.2
.4
1978
1982
1986
1990
1994
1998
2002
Year
Census
VR
CSS
DSS
Madagascar
Female
Male
$_{45}q_{15}$
0
.2
.4
.6
1978
1982
1986
1990
1994
1998
2002
Year
Census
VR
CSS
DSS

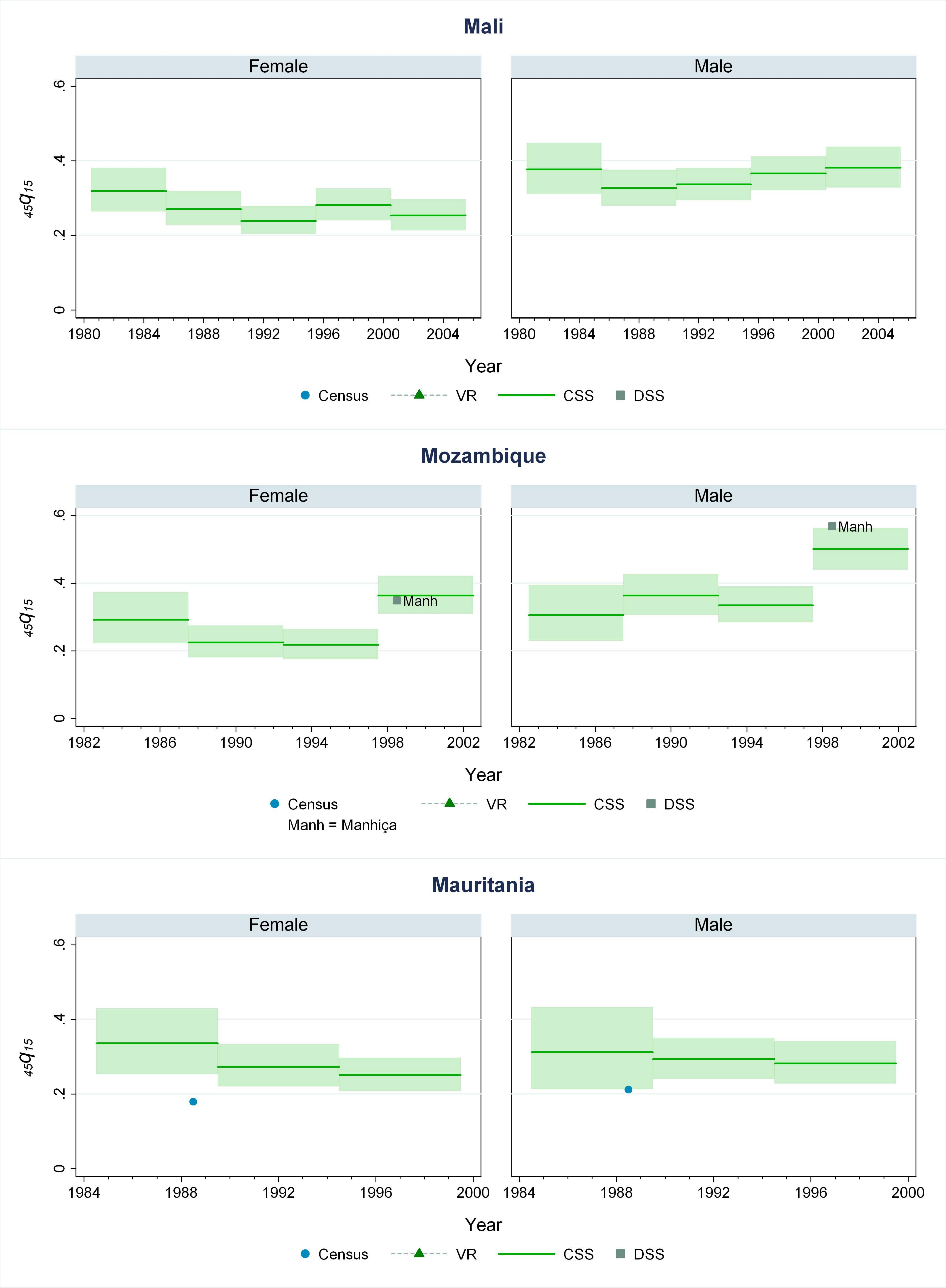

Mali
Female
Male
$_{45}q_{15}$
0
.2
.4
.6
1980
1984
1988
1992
1996
2000
2004
Year
Census
VR
CSS
DSS
Mozambique
Female
Male
$_{45}q_{15}$
0
.2
.4
.6
1982
1986
1990
1994
1998
2002
Manh
Year
Census
VR
CSS
DSS
Manh = Manhiça
Mauritania
Female
Male
$_{45}q_{15}$
0
.2
.4
.6
1984
1988
1992
1996
2000
Year
Census
VR
CSS
DSS

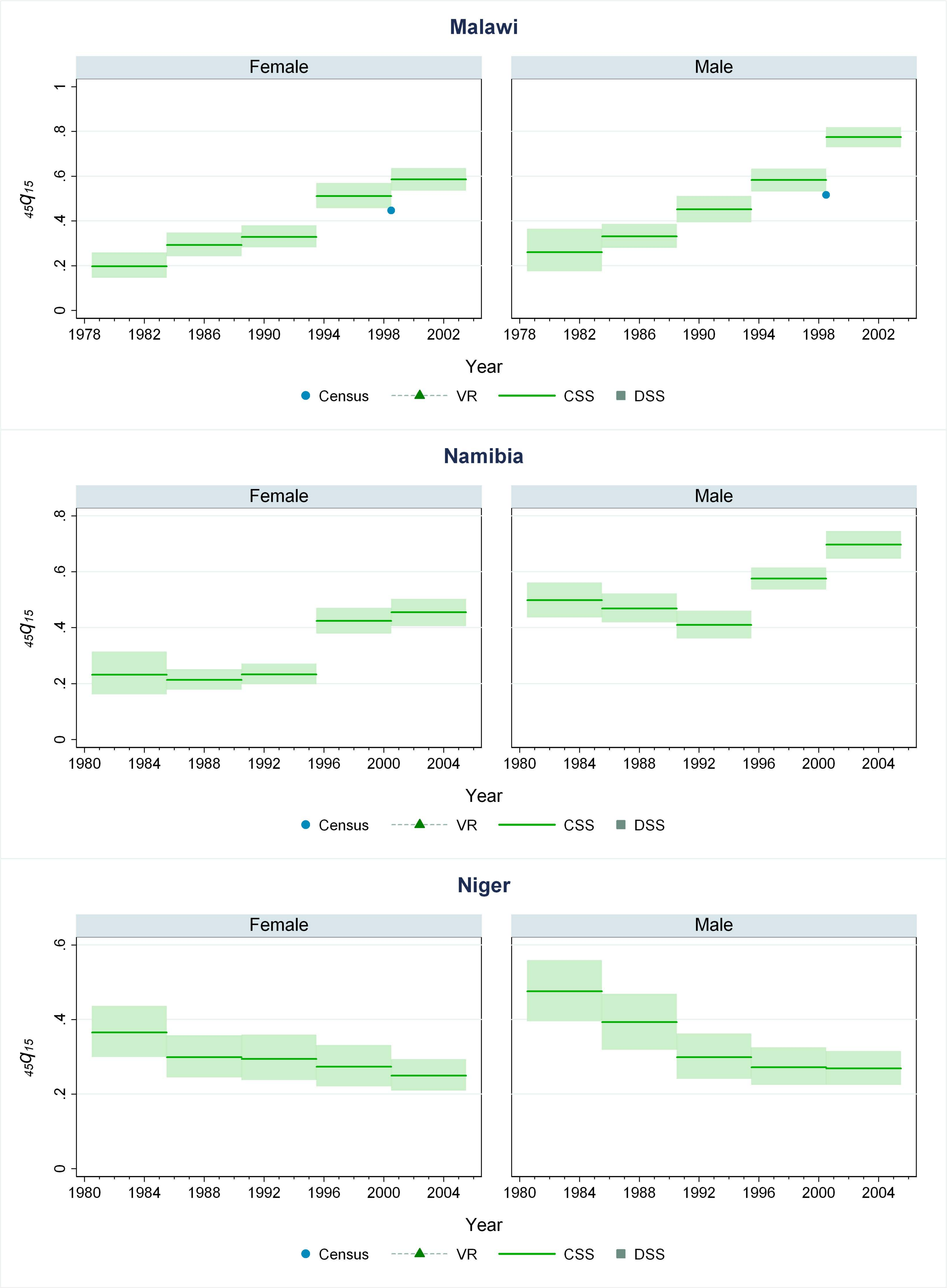

Malawi
Female
Male
$_{45}q_{15}$
0
.2
.4
.6
.8
1
1978
1982
1986
1990
1994
1998
2002
Year
Census
VR
CSS
DSS
Namibia
Female
Male
$_{45}q_{15}$
0
.2
.4
.6
.8
1980
1984
1988
1992
1996
2000
2004
Year
Census
VR
CSS
DSS
Niger
Female
Male
$_{45}q_{15}$
0
.2
.4
.6
1980
1984
1988
1992
1996
2000
2004
Year
Census
VR
CSS
DSS

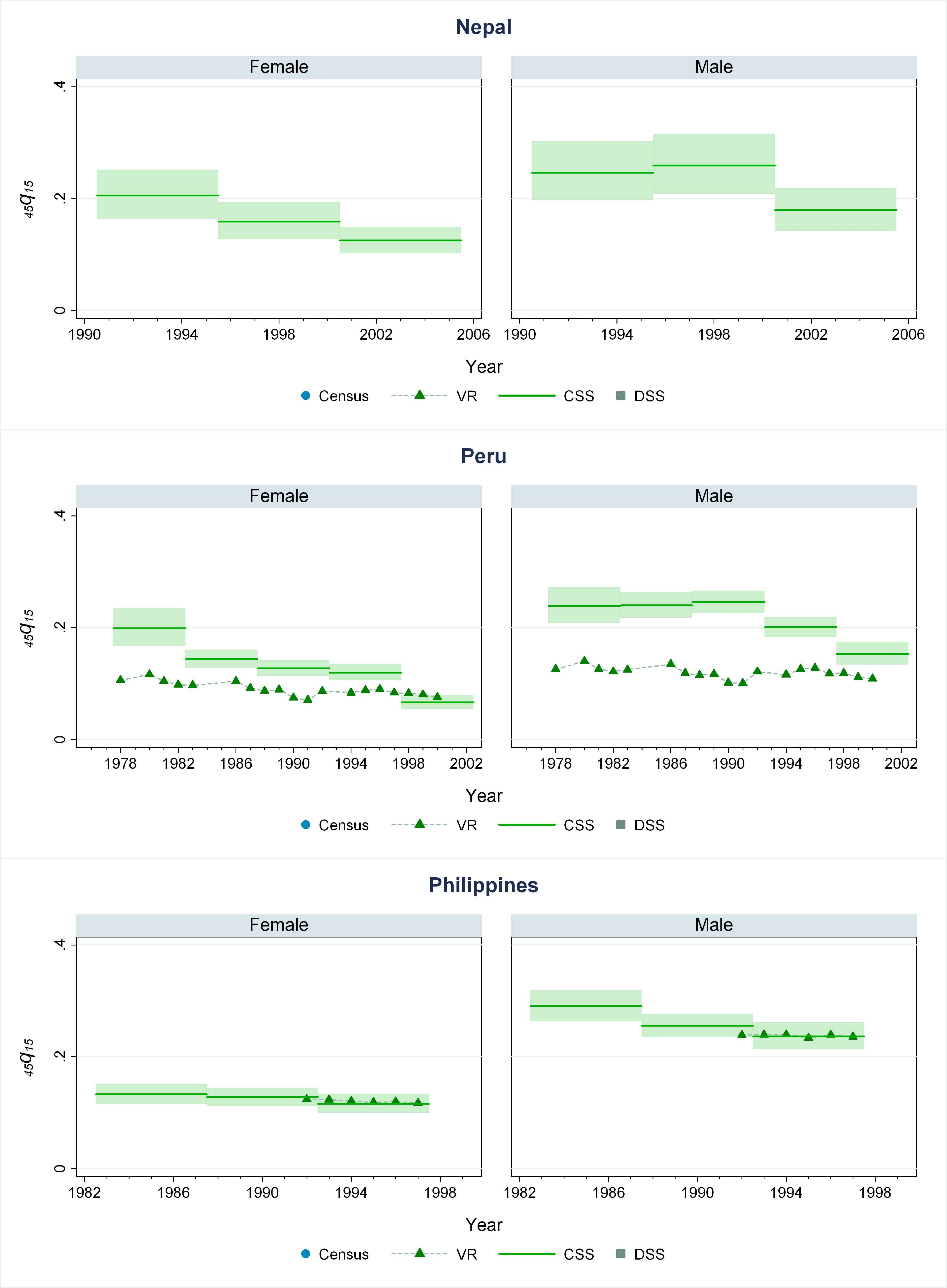
Nepal
Female
Male
0
.2
.4
$_{45}q_{15}$
1990
1994
1998
2002
2006
Year
Census
VR
CSS
DSS
Peru
Female
Male
0
.2
.4
$_{45}q_{15}$
1978
1982
1986
1990
1994
1998
2002
Year
Census
VR
CSS
DSS
Philippines
Female
Male
0
.2
.4
$_{45}q_{15}$
1982
1986
1990
1994
1998
Year
Census
VR
CSS
DSS

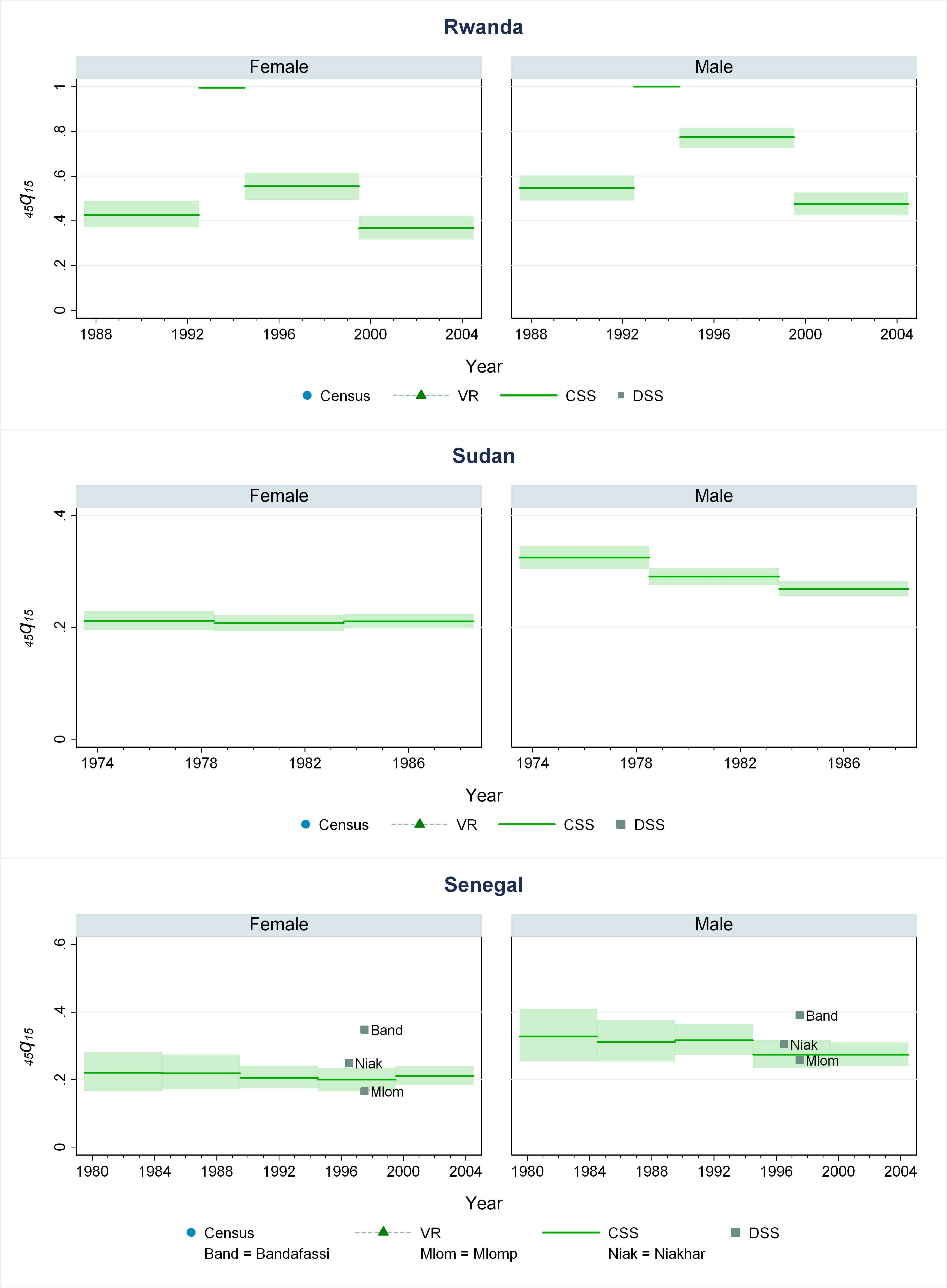
Rwanda
Female
Male
$_{45}q_{15}$
0
.2
.4
.6
.8
1
1988
1992
1996
2000
2004
Year
Census
VR
CSS
DSS
Sudan
Female
Male
$_{45}q_{15}$
0
.2
.4
1974
1978
1982
1986
Year
Census
VR
CSS
DSS
Senegal
Female
Male
$_{45}q_{15}$
0
.2
.4
.6
Band
Niak
Mlom
1980
1984
1988
1992
1996
2000
2004
Year
Census
VR
CSS
DSS
Band = Bandafassi
Mlom = Mlomp
Niak = Niakhar

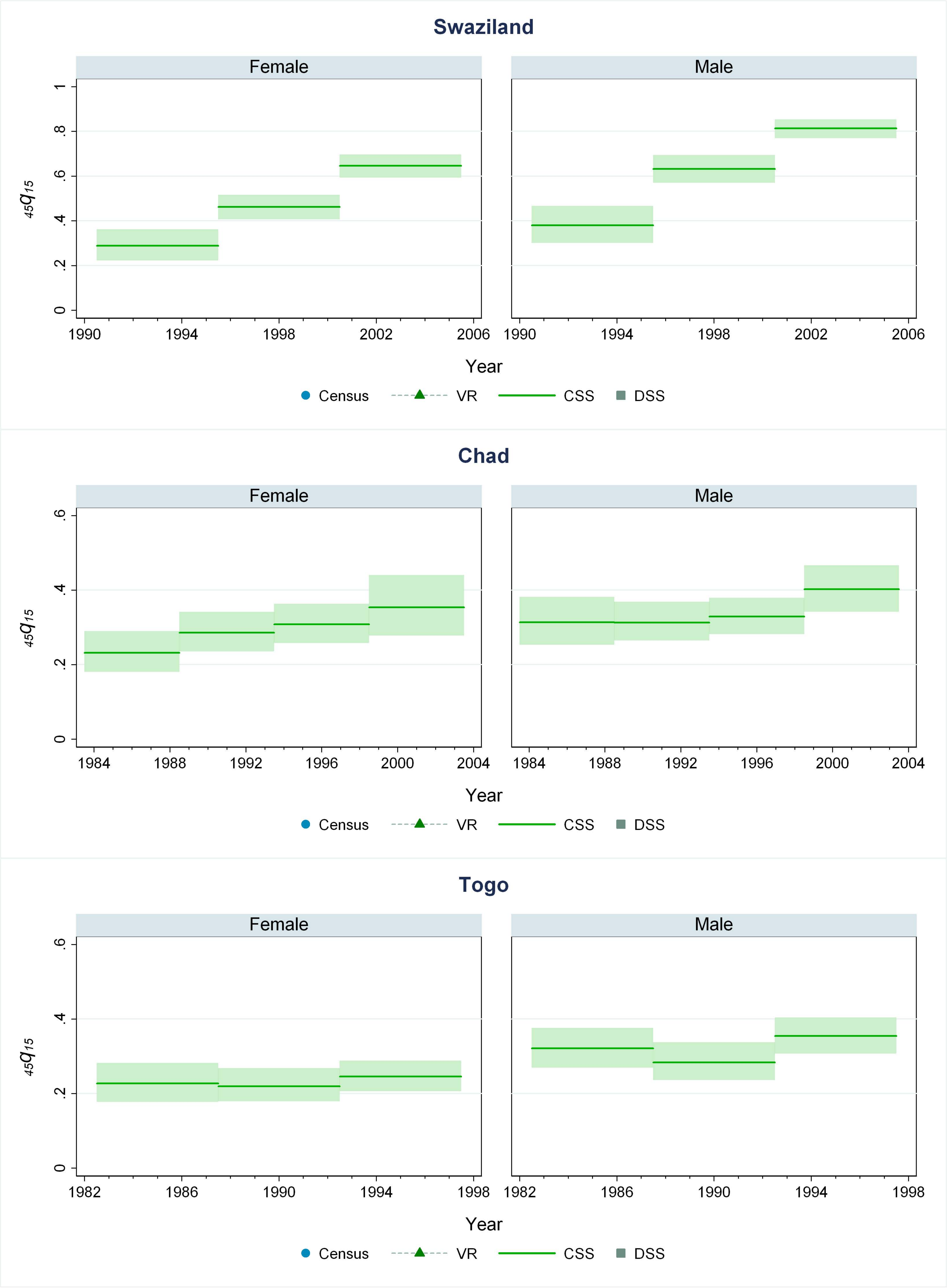
Swaziland
Female
Male
$_{45}q_{15}$
0
.2
.4
.6
.8
1
1990
1994
1998
2002
2006
Year
Census
VR
CSS
DSS
Chad
Female
Male
$_{45}q_{15}$
0
.2
.4
.6
1984
1988
1992
1996
2000
2004
Year
Census
VR
CSS
DSS
Togo
Female
Male
$_{45}q_{15}$
0
.2
.4
.6
1982
1986
1990
1994
1998
Year
Census
VR
CSS
DSS

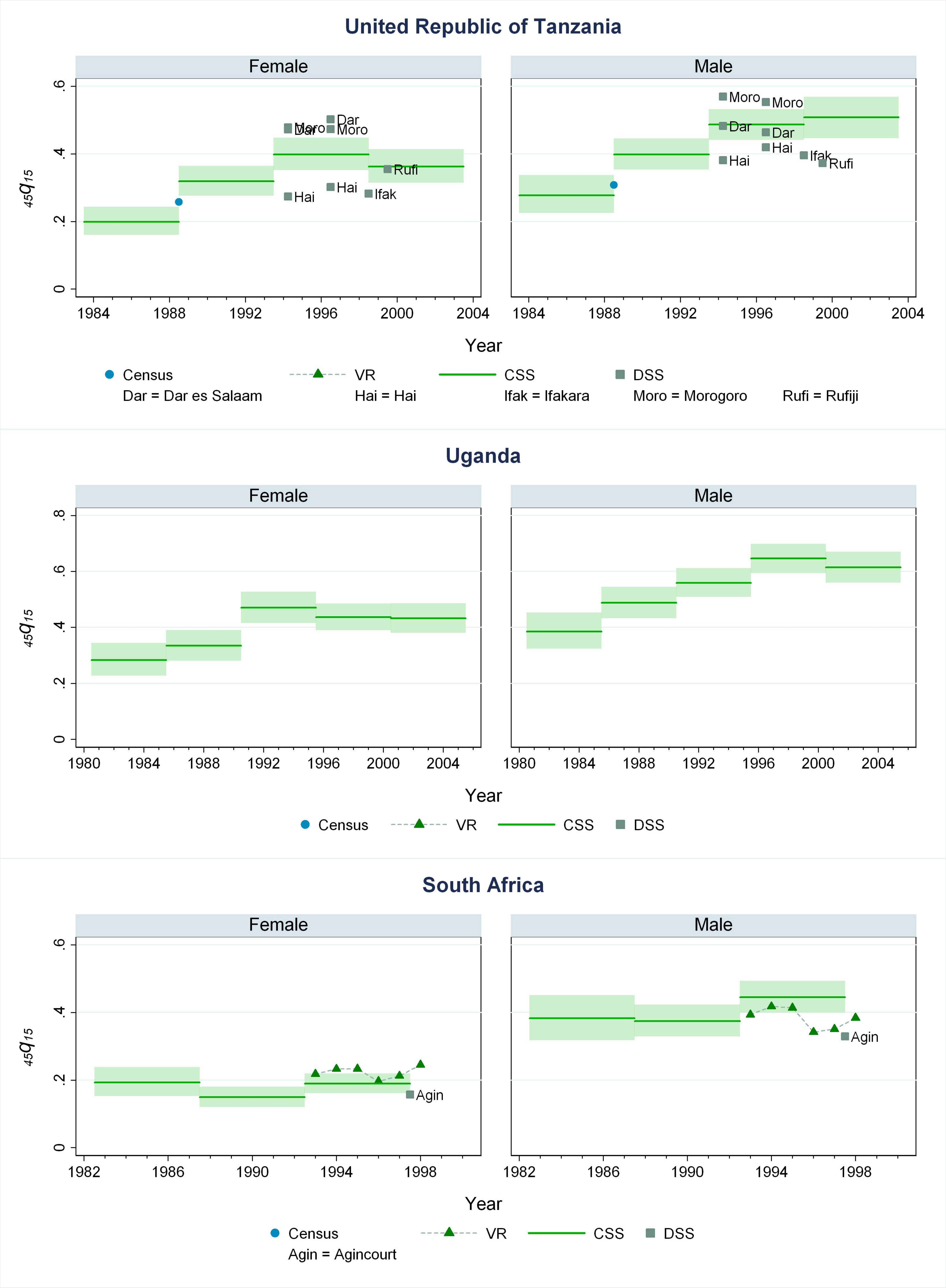
United Republic of Tanzania
Female
Male
.6
.4
.2
0
$_{45}q_{15}$
Moro
Dar
Hai
Ifak
Rufi
1984
1988
1992
1996
2000
2004
Year
Census
VR
CSS
DSS
Dar = Dar es Salaam
Hai = Hai
Ifak = Ifakara
Moro = Morogoro
Rufi = Rufiji
Uganda
Female
Male
.8
.6
.4
.2
0
$_{45}q_{15}$
1980
1984
1988
1992
1996
2000
2004
Year
Census
VR
CSS
DSS
South Africa
Female
Male
.6
.4
.2
0
$_{45}q_{15}$
Agin
1982
1986
1990
1994
1998
Year
Census
VR
CSS
DSS
Agin = Agincourt

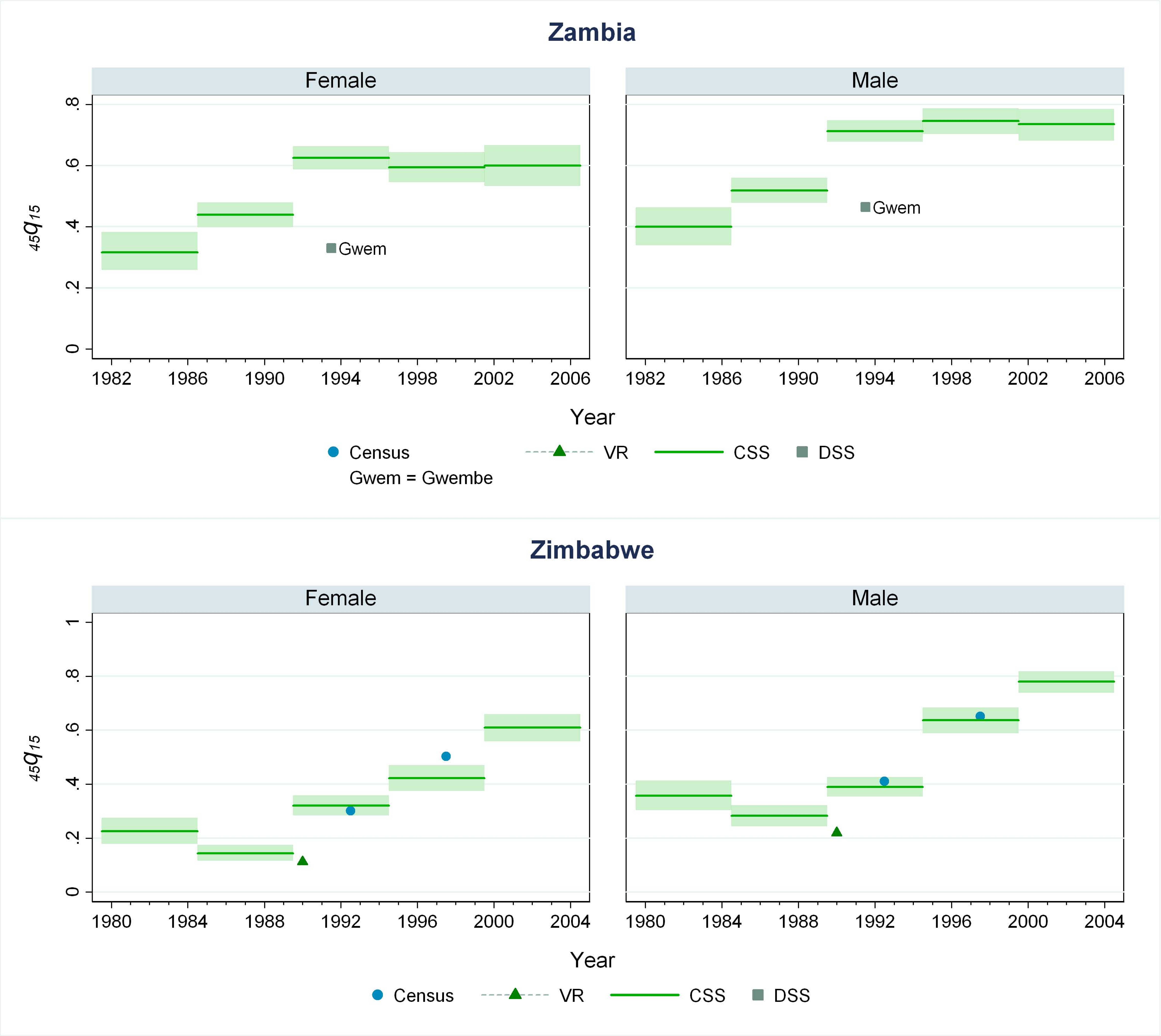

Zambia
Female
Male
Gwem
Gwem
.8
.6
.4
.2
0
1982
1986
1990
1994
1998
2002
2006
$_{45}q_{15}$
Year
Census
VR
CSS
DSS
Gwem = Gwembe
Zimbabwe
Female
Male
1
.8
.6
.4
.2
0
1980
1984
1988
1992
1996
2000
2004
$_{45}q_{15}$
Year
Census
VR
CSS
DSS
