# Supplementary material for: Measuring Adult Mortality Using Sibling Survival: A New Analytical Method and New Results for 44 Countries, 1974–2006
Source: PLoS Med. 2010 Apr 13;7(4):e1000260. doi: 10.1371/journal.pmed.1000260 (PMC2854132; doi:10.1371/journal.pmed.1000260)
Supplement: Text S1 — Derivation of zero-female-survivor correction. (0.03 MB PDF) [file pmed.1000260.s006.pdf]

Supporting Information Text S1: Derivation of zero-female-survivor correction

In order to calculate the correction factor to account for the lack of representation of families with no female survivors, we employed the following procedure. We can compute algebraically the undercount of female deaths for sibships of size *k* (assuming that mortality risks are constant across all sibships of size *k*):

$$D_k^{obs} = \pi_k \cdot N_k \cdot k - {\pi_k}^k \cdot N_k \cdot k$$

where D_k^{obs} is the number of female deaths observed in sibships of size *k* females, π_k is the true percent females dead in sibships of size *k*, and N_k is the total number of sibships of size *k* females. Therefore, in the equation above, $\pi \cdot N_k \cdot k$ is the true number of deaths in sibships of size *k*, and ${\pi_k}^k \cdot N_k \cdot k$ is the number of female deaths in sibships of size *k* females which are not observed because these deaths occur in sibships where all females die.

The observed percent of females who have died in sibships of size *k* females is represented by π_{obs} and is equal to the number of deaths observed in sibships of size *k* divided by the total number of females observed in sibships of size *k* (N_k^{obs} is the total number of sibships of size *k* females which we observe):

$$\pi_{obs} = \frac{D_k^{obs}}{N_k^{obs} * k}$$

Denoting N_k as the total number of sibships of size *k* females, then $N_k^{obs} = N_k - \pi_k{}^k * N_k$.

By substituting for D_k^{obs} and N_k^{obs}, we then arrive at an expression equating the observed percent dead with the true percent dead:

$$\pi_{obs} = \frac{D_k^{obs}}{N_k^{obs} * k} = \frac{\pi * N_k * k - \pi^k * N_k * k}{\left(N_k - \pi^k * N_k\right) * k} = \frac{\left(\pi - \pi^k\right) N_k * k}{\left(1 - \pi^k\right) N_k * k} = \frac{\left(\pi - \pi^k\right)}{\left(1 - \pi^k\right)}$$

While the mathematical solutions for π are infinite if $k = 1$, they are relatively easy to compute if $k = 2$ or 3. When $k > 3$, the solutions become quite complex. Fortunately, as sibship size increases, the contribution to total sibships of size *k* females of sibships where all females have died becomes substantively insignificant. Therefore, we use the algebraic solutions to correct the percent dead for sibship sizes 2 and 3 and then assume a linear relationship between (corrected) percent dead and sibship size to predict the true percent dead for sibships of only 1 female. In Appendix Figure 1, the uncorrected and corrected percent dead is shown by sibship size for males and females, using an example from the Mali 2005 DHS. Using these corrected percentages and the observed number of deaths for each sibship size, we compute the percent of total female deaths which are not captured due to the zero-surviving-female phenomenon. Finally, we correct the CSS estimates of age specific probabilities of death upward by this factor and recompute ${}_{45}q_{15}$.
